# Dual‐Gating Strategy: Ultrasound Activation of TRPV2 Channels and Borate‐Glass‐Induced Calcium Overload for Tumor Suppression

**DOI:** 10.1002/advs.202414676

**Published:** 2025-02-27

**Authors:** Haihong Zhu, Liping Ouyang, Yangguang Huang, Ji Tan, Chunyu Liu, Qian Wang, Rongkun Huang, Wing Tak Wong, Xuanyong Liu, Haobo Pan, Yun Liao

**Affiliations:** ^1^ Department of Pharmacy, Shanghai General Hospital Shanghai Jiao Tong University School of Medicine Shanghai 200080 China; ^2^ Department of Pharmacy, Tongren Hospital Shanghai Jiao Tong University School of Medicine Shanghai 200336 China; ^3^ Hongqiao International Institute of Medicine Shanghai Jiao Tong University School of Medicine Shanghai 200336 China; ^4^ State Key Laboratory of Advanced Ceramics Shanghai Institute of Ceramics Chinese Academy of Sciences Shanghai 200050 China; ^5^ Shenzhen Key Laboratory of Marine Biomedical Materials CAS‐HK Joint Lab of Biomaterials, The Key Laboratory of Biomedical Imaging Science and System, Shenzhen Institute of Advanced Technology Chinese Academy of Sciences Shenzhen 518055 China; ^6^ School of Life Sciences Faculty of Science The Chinese University of Hong Kong Hong Kong 999077 China; ^7^ School of Chemistry and Materials Science Hangzhou Institute for Advanced Study University of Chinese Academy of Sciences Sub‐Lane Xiangshan Hangzhou 310024 China

**Keywords:** borate glass, calcium overload, precise breast cancer treatment, TRPV2, ultrasound

## Abstract

Effective and precise treatment of breast cancer, particularly with bone metastasis, remains a significant challenge. Here, a dual‐gating strategy combining locally delivered borate glass (BG) and ultrasound (US) is developed for the precise and effective inhibition of breast cancer by targeting transient receptor potential vanilloid 2 (TRPV2). The results demonstrate that after local delivery of BG to the solid tumor, US effectively triggers calcium overload by activating the overexpressed TRPV2 channels, leading to mitochondrial autophagy and apoptosis in breast cancer cells, thereby inhibiting tumor growth with high precision. These effects are validated in subcutaneous, orthotopic, and TRPV2‐overexpressing breast cancer mouse models. In the bone metastasis model, BG combined with US treatment simultaneously suppresses tumor growth and promotes bone regeneration. Overall, this dual‐gating strategy offers a safe and efficient approach for the precise treatment of cancers with high TRPV2 expression and provides new insights into the design and clinical translation of calcium‐overload‐based cancer therapies.

## Graphical abstract

A novel strategy combining borate glass and ultrasound is proposed for the precise treatment of breast cancer. The locally delivered borate glass releases Ca^2^⁺ ions in situ, while ultrasound activates the highly expressed transient receptor potential vanilloid 2 channels in breast cancer cells. This synergistic effect induces specific calcium overload, leading to targeted cell apoptosis.

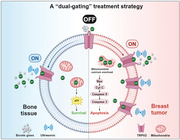

## 1 Introduction

Breast cancer is the most common malignancy in women and the second leading cause of cancer‐related mortality globally.^[^ ^1^ ^]^ Traditional therapies such as surgery, chemotherapy, and endocrine therapy are limited by drug resistance, systemic toxicity, and poor specificity.^[^ ^2^ ^]^ Targeted therapies, including those focused on estrogen receptors, Epidermal Growth Factor Receptor 2, and Cyclin‐Dependent Kinase4/6, struggle with tumor heterogeneity and complex microenvironments, particularly in triple‐negative breast cancer (TNBC).^[^ ^3^ ^]^ These challenges highlight the urgent need for innovative, precise treatment options.

Calcium overload, defined by the excessive accumulation of Ca^2^⁺ in the cytoplasm, has emerged as a promising strategy for cancer therapy.^[^ ^4^ ^]^ Prolonged calcium overload can induce mitochondrial dysfunction, Reactive Oxygen Species (ROS) generation, plasma membrane damage, and immune responses, ultimately triggering apoptosis in cancer cells.^[^ ^5^ ^]^ Recently, the potential of calcium‐overload‐based therapy has been explored as an anti‐breast‐cancer strategy using calcium‐containing nanomaterials.^[^ ^6^ ^]^ However, these studies fall short of the requirements for precise therapy due to the significant off‐target effect of calcium‐based nanomaterials. Various calcium‐containing nanodevices based on calcium phosphate, CaCO₃, CaO₂, CaH₂, and CaS, have been developed to suppress tumors through the calcium overload mechanism.^[^ ^7^ ^]^ However, their nanoscale nature presents challenges, as these materials can be easily internalized by nontumor cells both during circulation and at the tumor site, potentially disrupting calcium signaling in healthy cells and raising concerns about their biosafety.^[^ ^8^ ^]^

Micrometer‐sized calcium sources that can be locally delivered to maintain high Ca^2^⁺ levels at tumor sites offer a promising alternative. The micrometer‐sized materials (especially significantly larger than cells) cannot be readily internalized by cells, avoiding the unintended effects of nanosized materials. However, few micrometer‐sized calcium‐based materials have been developed for this purpose. Calcium‐containing borate glass (BG) is a novel biomedical ceramic with several advantages, including excellent biocompatibility, fast biodegradability, and potent tissue regeneration capabilities (e.g., for bone and soft tissues).^[^ ^9^ ^]^ Unlike traditional silicate bioactive glass, BG degrades more readily in aqueous environments due to the cleavage of B─O bonds, releasing Ca^2^⁺ during degradation. Furthermore, micrometer‐sized BG powder can be efficiently produced at a large scale using the melt‐quenching method.^[^ ^9b^ ^]^ These characteristics position BG as a promising locally deliverable calcium source with dual functions: promoting tissue repair and suppressing tumor growth. This dual functionality could be particularly advantageous in treating bone metastasis of breast cancer.

Inducing specific Ca^2^⁺ influx and accumulation in breast cancer cells, while sparing noncancerous cells, is key to precise calcium‐overload‐based treatments. Transient receptor potential vanilloid 2 (TRPV2), a calcium‐permeable ion channel, plays a crucial role in regulating intracellular calcium levels and is notably upregulated in breast cancer cells, making it a potential target for inducing calcium overload.^[^ ^10^ ^]^ However, the influx of Ca^2^⁺ is precisely controlled by cells, requiring external stimuli to activate TRPV2. TRPV2 can be activated by thermal, oxidative, and pharmacological agents, but these methods may cause collateral tissue damage or toxicity.^[^ ^11^ ^]^ Ultrasound (US) offers a promising alternative due to its noninvasive nature, deep tissue penetration, and ability to precisely control spatial and temporal stimulation.^[^ ^12^ ^]^ US can enhance tumor barrier permeability, facilitating drug delivery, and, due to the mechanosensitivity of TRPV2, has the potential to activate TRPV2, further promoting targeted calcium overload in breast cancer cells.^[^ ^13^ ^]^

Based on the above statement, herein, a dual‐gating strategy combining locally delivered BG and US was developed to precisely inhibit breast cancer by targeting TRPV2. In this approach, BG creates a Ca^2^⁺‐enriched microenvironment, while US activates the TRPV2 channel and accelerates Ca^2^⁺ release. This results in efficient calcium overload in breast cancer cells with high TRPV2 expression, while minimizing effects on cells with low TRPV2 expression, thereby enabling targeted inhibition. Additionally, BG promotes bone regeneration in cases of breast cancer metastasis to bone. With BG's excellent biosafety profile and US's clinical approval, this dual‐gating strategy offers a safe and efficient method for the precise treatment of cancers with high TRPV2 expression, providing new insights into the design of calcium‐overload‐based cancer therapies.

## 2 Results

### 2.1 Characterization of BG and the Influence of Ultrasound on Its Properties

**Figure 1:**
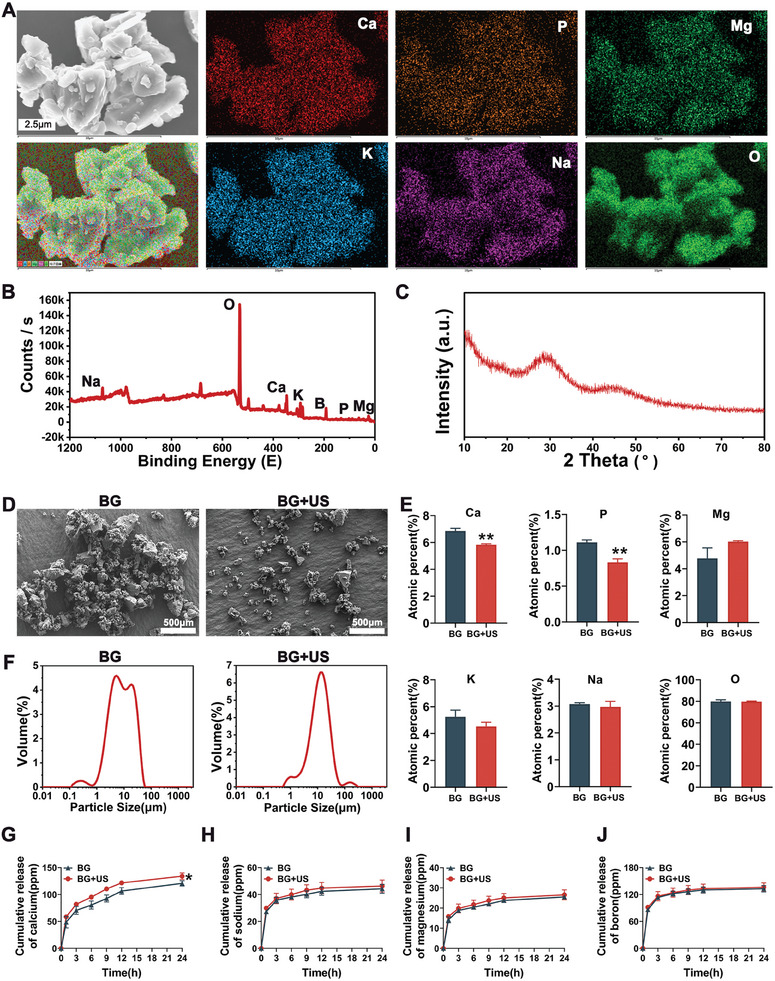
Characterization of BG and the influence of ultrasound on its properties. A) EDS spectrum of the BG material, featuring SEM images of the test area and elemental maps for calcium, phosphorus, magnesium, potassium, sodium, and oxygen. B) Full‐scan XPS spectrum of the BG material. C) XRD pattern of BG. D) The morphological characterization of BG with or without US treatment by SEM. E) Statistical analysis of elemental content changes in the EDS spectrum pre‐ and post‐US treatment. F) Changes in BG particle size pre‐ and post‐US treatment. G) Calcium ion release curve for BG dispersed in deionized water, with or without US intervention. H) Sodium ion release curve. I) Magnesium ion release curve. J) Boron ion release curve. Data are presented as mean ± standard deviation (SD) (*n* = 3). Statistical significance was determined using a *t*‐test. *: compared to BG group, *p < 0.05, **p < 0.01.

The elemental composition of the BG material was analyzed using energy dispersive X‐ray spectroscopy (EDS), revealing the presence of Ca, P, Mg, K, Na, and O (**Figure** **1**A). X‐ray photoelectron spectroscopy (XPS) was employed to identify the composition of BG. The results showed that there were Na, Ca, Mg, K, O, B, and P (Figure 1B). Furthermore, X‐ray diffraction (XRD) analysis validated that the XRD pattern of BG primarily exhibits a broad diffuse peak, lacking sharp crystalline diffraction peaks, which is characteristic of amorphous materials, as illustrated in Figure 1C. A comparative study of BG dispersed in anhydrous ethanol, analyzed using scanning electron microscopy (SEM), demonstrated that US intervention significantly enhanced BG dispersion compared to the non‐US group (Figure 1D). Elemental content was measured via EDS before and after US treatment, showing a notable increase in the release of calcium and phosphorus ions from the BG material following US exposure (Figure 1E). Particle size measurements conducted using the Zetatrac particle size analyzer revealed that US treatment led to a more uniform particle size distribution, with a range from 3.899 to 32.540 µm (Figure 1F). Additionally, the ion release from BG dispersed in deionized water with or without US treatment was assessed using inductively coupled plasma (ICP). Figure 1G–J and Figure S1 (Supporting Information) present the cumulative release curves of calcium, sodium, magnesium, and boron ions over 24 h, indicating that US significantly promoted calcium ion release. In summary, these results demonstrate that US effectively enhances the dispersion of BG, resulting in a more uniform particle size distribution and significantly increasing ion release from the material.

### 2.2 BG Combined with US for Effective and Precise Inhibition of Breast Cancer Cells

**Figure 2:**
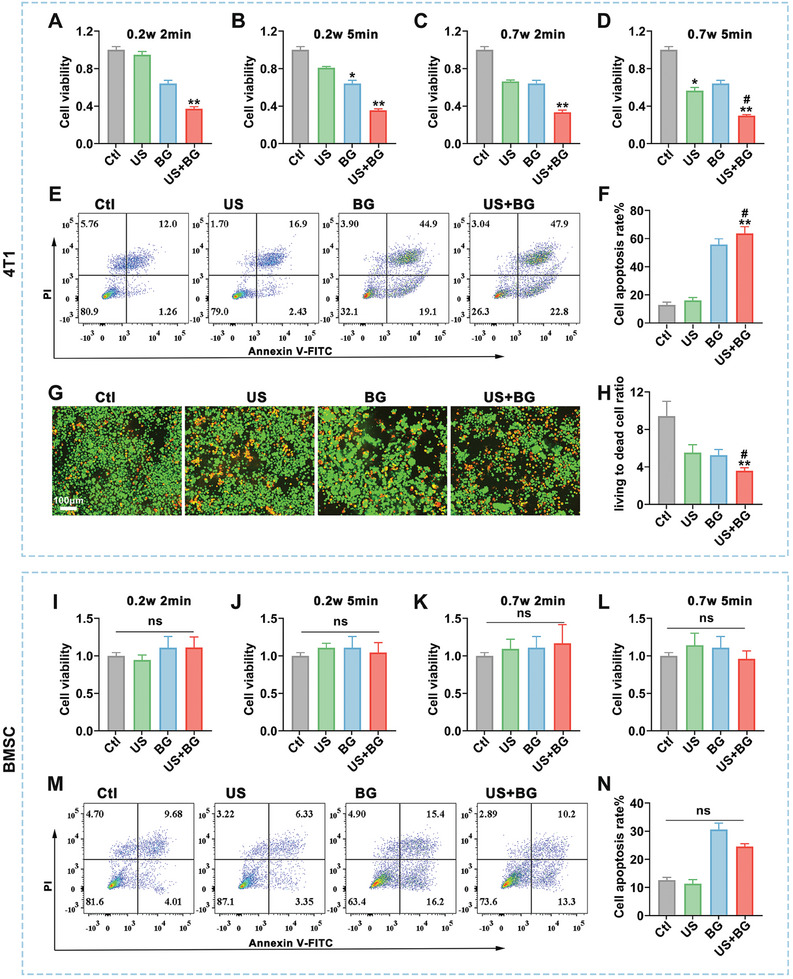
BG combined with US for effective and precise inhibition of breast cancer cells. The viability of 4T1 cells incubated with 1 mg mL^−1^ BG and treated with US of variable intensity and duration: A) 0.2 W, 2 min; B) 0.2 W, 5 min; C) 0.7 W, 2 min; D) 0.7 W, 5 min. E) Flow cytometry analysis of apoptosis rate of 4T1 cells in each group (US 0.7 W, 5 min). F) Statistical results of the apoptosis rate in 4T1 cells (US 0.7 W, 5 min). G) Live/dead cell staining of 4T1 cells in each group (US 0.7 W, 5 min). H) Statistical results of live/dead staining. The viability of BMSC incubated with 1 mg mL^−1^ BG and treated with US of variable intensity and duration: I) 0.2 W, 2 min; J) 0.2 W, 5 min; K) 0.7 W, 2 min; L) 0.7 W, 5 min. M) Flow cytometry analysis of apoptosis rate of BMSC in each group (US 0.7 W, 5 min). N) Statistical data of apoptosis rate of BMSC. Data were presented as mean ± SD (*n* = 3). Statistical significance was assessed by one‐way ANOVA followed by Tukey's multiple comparison tests. *: compared with the control group, *p < 0.05, **p < 0.01; #: compared with the BG group, #*p* < 0.05.

The analysis of US intensity distribution revealed an effective US energy in the *Z* direction (Figure S2, Supporting Information), ensuring its application to the cells in plates. To explore the impact of US intensity and duration on the proliferation of breast cancer cells, the cell counting kit‐8 (CCK‐8) assay was employed to assess cell viability under various US conditions. The results indicated that US inhibited breast cancer cell proliferation in a time‐ and intensity‐dependent manner. As US intensity increased, the inhibition rate also rose, with the most potent effect observed at 0.7 W for 5 min, where cell viability was reduced to 56.5% (Figure S3A, Supporting Information). To determine the optimal BG concentration for combined treatment, we evaluated the effect of different BG concentrations on breast cancer cell proliferation. The results showed a concentration‐dependent inhibition within the 0–1 mg mL^−1^ range. BG exhibits the strongest inhibitory effect with 1 mg mL^−1^, reducing cell viability to 64%. However, further increasing the concentration reversed this effect (Figure S3B, Supporting Information). We further assessed the impact of various intervals (0, 3, 6, 12, 24 h) between the addition of BG and the subsequent US intervention on cell viability. The results demonstrated that initiating the US intervention 6 h after the addition of BG produced the most significant therapeutic effect (Figure S3C, Supporting Information). Based on these findings, 1 mg mL^−1^ BG with US intervention 6 h after the addition of BG was selected for further studies. Under the condition of 2 min with 0.2 W US, the BG combined with US treatment group had a cell viability of 37.2% (**Figure** **2**A). As US intensity and duration increased, cell viability of the combined group further decreased, reaching 29.8% under 5 min irradiation with 0.7 W US (Figure 2B–D). Therefore, we selected the combined condition of 0.7 W US for 5 min for the subsequent experiments. The flow cytometry was used to detect the impact of the combined treatment on breast cancer cell apoptosis. The results revealed that BG alone promoted apoptosis (55.8%), and this effect was significantly enhanced with the additional treatment of US (63.4%) (Figure 2E,F). To more clearly observe the survival status of the cells, a live/dead cell staining was performed. The results demonstrated that the BG combined with US treatment exhibited the lowest live/dead cell ratio among the four groups. Furthermore, the BG combined with US treatment significantly increased the cell apoptosis ratio and reduced the live cell amounts compared to the BG‐only group (Figure 2G,H). Additionally, the inhibitory effects of BG combined with US were evaluated in another TNBC cell line, MDA‐MB‐231. Cell viability and apoptosis assays demonstrated that BG combined with US significantly inhibited proliferation and induced apoptosis in MDA‐MB‐231 cells 24 h after treatment (Figure S4, Supporting Information). These results suggest the broad applicability of our strategy for anti‐breast‐cancer treatment.

To assess the tolerability of normal cells to this combined treatment, the effects on bone marrow mesenchymal stem cells (BMSCs) were detected. The results showed that BMSC maintained well viability across four US intensities, and both US and BG promoted BMSC proliferation to varying degrees (Figure 2I–L). Importantly, neither BG alone nor combined with US significantly promoted apoptosis in BMSC (Figure 2M,N). Collectively, these findings suggest that BG combined with US exhibits superior effects in inhibiting proliferation and promoting apoptosis in breast cancer cells, without exhibiting similar effects in normal BMSC.

### 2.3 The Antitumor Effects of BG Combined with US in Subcutaneous and Orthotopic Tumor Models

**Figure 3:**
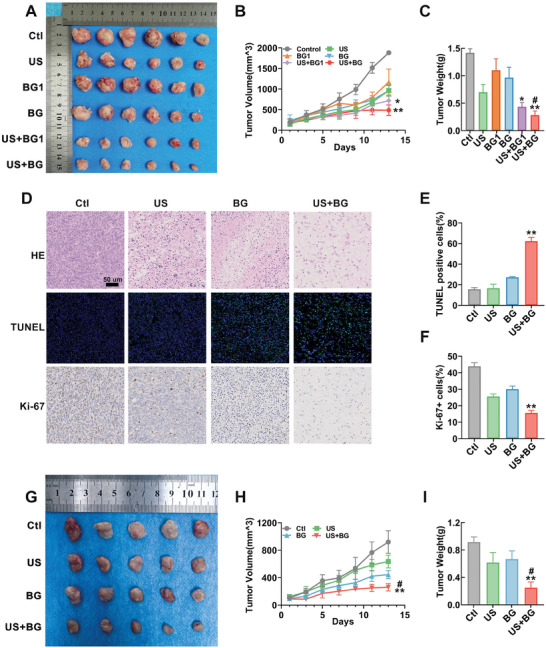
The antitumor effects of BG combined with US in subcutaneous and orthotopic tumor models. A) The photograph of extracted subcutaneous tumors. BG1 group: mice treated with 1 mg BG; BG group: mice treated with 5 mg BG; US+BG1 group: mice treated with 1 mg BG and US; US+BG group: mice treated with 5 mg BG and US. B) The growth curve of dynamic tumor volumes of the samples in each group. C) Statistical results of subcutaneous tumor weights posttreatment. D) Representative immunohistochemical images of subcutaneous tumors, including H&E staining, TUNEL staining for apoptosis, and Ki67 staining for proliferation. Quantitative analysis of E) TUNEL and F) Ki67 staining. G) Gross image of the orthotopic breast tumors in BALB/c mice after different treatment interventions. H) The growth curve of dynamic tumor volumes of the samples in each group. I) Statistical results of orthotopic tumor weights posttreatment. Data are presented as mean ± SD (*n* = 5). Statistical significance was determined using one‐way ANOVA followed by Tukey's multiple comparison tests. *: compared with the control group, *p < 0.05, **p < 0.01; #: compared with the BG group, #*p* < 0.05.

To further investigate the in vivo antitumor efficacy of US combined with BG, both subcutaneous and orthotopic breast cancer models of mouse were established. The subcutaneous breast cancer animal model experimental design is outlined in Figure S5 (Supporting Information). The body weight monitoring revealed no significant differences among these groups, indicating the treatment acceptability (Figure S6A, Supporting Information). 15 days after the first intervention, the solid tumor tissues in all groups were extracted for gross observation. As shown in **Figure** **3**A, all intervention groups showed a decrease in tumor size compared to the control group, with the US+BG1 (1 mg dose) and US+BG (5 mg dose) groups showing the most pronounced tissue shrinkage. Tumor volume progression was also recorded. Figure 3B illustrates the tumor volume trend in each group. In the control group, the tumor growth progressed the fastest due to the lack of intervention. In the US, BG1, BG, and US+BG1 groups, tumor growth was slower than in the control group, but continued to progress. However, in the US+BG group, tumor growth was successfully terminated at 9 days, demonstrating the highest efficacy in suppressing tumor progression. Figure 3C shows the tumor tissue weight for each group. Correspondingly, the tumor samples in US+BG group exhibit the lightest weight. Collectively, these results demonstrate that the US+BG (5 mg dose) intervention effectively inhibited breast cancer progression in the subcutaneous model, and this dose was selected for subsequent in vivo experiments. To assess tumor cell proliferation and apoptosis, histological and immunohistochemical analyses were performed on the samples of the control, US, BG, and US+BG groups. Representative images of hematoxylin and eosin (H&E) staining, terminal deoxynucleotidyl transferase dUTP nick end labeling (TUNEL) staining (apoptotic cells), and KI67 staining (proliferative cells) are shown in Figure 3D. Quantitative analysis of TUNEL‐positive cells (Figure 3E) and KI67‐positive cells (Figure 3F) further elucidated the impact of BG in combination with US on tumor cell apoptosis and proliferation.

Motivated by the remarkable antitumor effects of the combined intervention method observed in subcutaneous tumor models, we undertook the injection of 4T1 cells into the mammary fat pad beneath the third mammary gland of mice to establish an orthotopic breast cancer model, aiming to further confirm and validate the efficacy of this intervention approach. Figure 3G displays images of the orthotopic tumors posttreatment, demonstrating that the tumor size was significantly reduced in the BG combined with US irradiation group compared to the control group. Tumor volume and body weight changes during the treatment period were also monitored (Figure 3H and Figure S6B (Supporting Information)). The findings showed a significant tumor volume reduction in the BG with US irradiation group compared to both the control and BG alone groups. The weights of the orthotopic tumors after treatment were also recorded (Figure 3I). The results indicated that, in comparison to the control group, the tumor weights in the US group, BG group, and the BG combined with US irradiation group were all reduced, with the BG combined with US irradiation group exhibiting a significant reduction. The results demonstrated that US combined with BG effectively inhibits breast cancer growth and induces cell apoptosis in both orthotopic and subcutaneous tumor models. Subsequently, the calcium distribution and biosafety of BG with or without US intervention were evaluated in the orthotopic model. H&E staining of the main organs (heart, liver, lung, and kidney) and tissues (skin and muscle) around the administration site showed similar results across the control, BG, and US+BG groups, with no significant tissue damage observed (Figure S7A,B, Supporting Information). Liver and kidney function tests confirmed normal function in the mice from both the BG and US+BG groups (Figure S7C–H, Supporting Information). As shown in Figure S7I–L (Supporting Information), after six treatment sessions, no significant changes in calcium levels were detected in the heart, liver, lung, and kidney. Overall, the dual‐gating strategy not only effectively inhibits breast cancer growth but also demonstrates low toxicity to normal tissues, highlighting its potential for clinical application.

### 2.4 Activating TRPV2 Channels with US for Enhancing Calcium Influx

**Figure 4:**
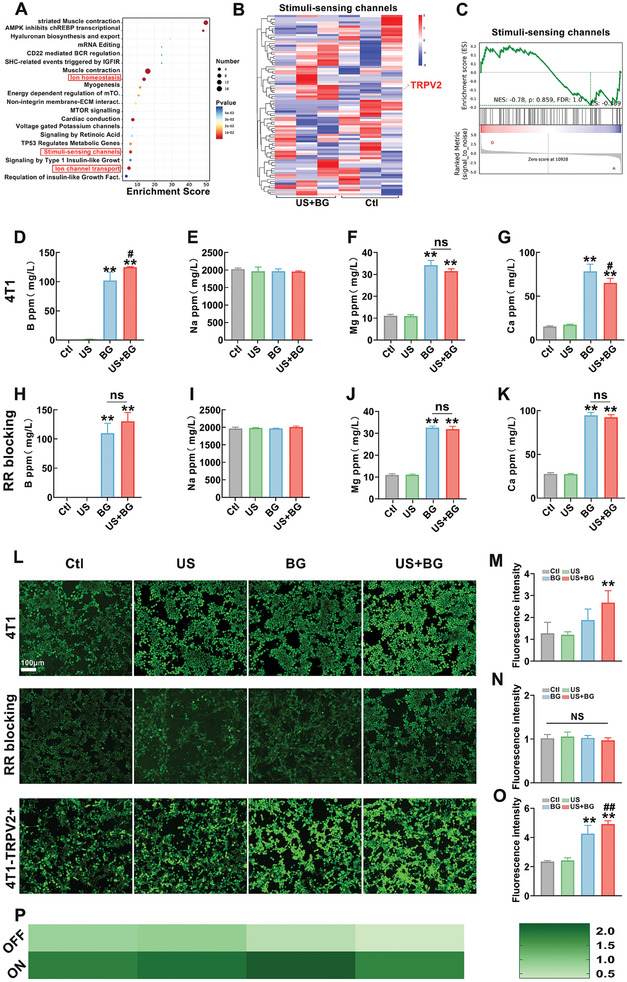
Activating TRPV2 channels with US for enhancing calcium influx. A) KEGG pathway enrichment analysis based on whole transcriptome sequencing. B) Heatmap illustrating gene expression profiles. C) GSEA analysis of stimuli‐sensing channel pathways. Statistical analysis of D) boron ions, E) Na^+^, F) Mg^2+^, and G) Ca^2+^ concentrations in the supernatant of 4T1 cells 24 h after treatments. Statistical analysis of H) boron ions, I) Na^+^, J) Mg^2+^, and K) Ca^2+^ concentrations in the supernatant of 4T1 cells preincubated with ruthenium red 24 h after treatments. L) Representative fluorescent images of intracellular calcium levels in normal 4T1 cells, ruthenium‐red‐preincubated 4T1 cells, and TRPV2‐overexpressing 4T1 cells 24 h after treatments. The intracellular levels were detected by Flow‐4 calcium assay methods, and the intensity of the green fluorescence is positively correlated with intracellular calcium level. M) Quantitative fluorescent intensity of intracellular calcium levels in normal 4T1 cells, N) ruthenium‐red‐preincubated 4T1 cells, and O) TRPV2‐overexpressing 4T1 cells 24 h after treatments. The fluorescent intensity was detected by microplate reader. P) Heatmap of fluorescent intensity ratio among normal 4T1 cells, ruthenium‐red‐treated 4T1 cells, and TRPV2‐overexpressing 4T1 cells. The data are presented as mean ± SD (*n* = 3). Statistical significance was assessed using one‐way ANOVA followed by Tukey's multiple comparison tests. *: compared with the control group, *p < 0.05, **p < 0.01; #: compared with the BG group, #*p* < 0.05; ##*p* < 0.01.

To elucidate the molecular mechanisms underlying the superior in vivo and in vitro antitumor effects observed with the combined treatment, we conducted whole‐transcriptome sequencing analysis from both the control group and the combination therapy group in the subcutaneous tumor model. Kyoto Encyclopedia of Genes and Genomes (KEGG) pathway enrichment analysis revealed significant enrichment of pathways related to ion homeostasis, stimulus‐responsive channels, and ion transport channels (**Figure** **4**A). A heatmap of genes associated with stimulus‐responsive channels indicated a significant upregulation of the *TRPV2* gene in the combination therapy group (Figure 4B), which was further corroborated by Gene Set Enrichment Analysis (GSEA) (Figure 4C). Additionally, qRT‐PCR analysis of TRPV2 expression in 4T1 cells cultured in vitro after treatment corresponded to the transcriptome sequencing results (Figure S8A, Supporting Information). Interestingly, the TRPV2 expression levels in BMSC were not affected by the treatment, indicating the potential of TRPV2 as a target for precise anti‐breast‐cancer therapy (Figure S8B, Supporting Information).

Previous studies have shown that TRPV2 can regulate the influx and efflux of calcium ions in cells, thereby affecting their function. Therefore, cell supernatants were collected after 24 h of different interventions, and the ion concentrations in the supernatants were measured. It was found that the concentrations of boron ions significantly increased after the BG combination with US and there was no significant difference in Na^+^ and Mg^2+^ between BG and US+BG group (Figure 4D–F). However, it is noteworthy that the concentration of Ca^2+^ in the cell supernatant significantly decreased in the US combined with BG group compared to the BG group alone (Figure 4G), suggesting that more calcium ions might have entered the cells by TRPV2. To confirm this hypothesis, breast cancer cells were incubated with a TRPV2 channel blocker for 1 h, then exposed to different interventions. After 24 h, the cell supernatants were collected again for ion concentration detection. The results showed that after blocking the TRPV2 channel, the concentrations of boron ions, Na^+^, and Mg^2+^ in the extracellular fluid of the US combined with BG group remained unchanged compared to before the blockade (Figure 4H–J). However, the previously decreased extracellular calcium levels were corrected, resulting in an increase in calcium concentration after the blockade compared to before the blockade (Figure 4K).

Next, the intracellular calcium ion concentration was directly measured by Fluo‐4AM staining in 4T1 cells, TRPV2‐blocked 4T1 cells (pretreated by ruthenium red), and TRPV2‐overexpressed 4T1 cells following US and BG interventions. Figure 4L shows representative fluorescent images of intracellular calcium ions in normal 4T1 cells, ruthenium‐red‐treated 4T1 cells, and TRPV2‐overexpressed 4T1 cells. The statistical results of fluorescent intensity (Figure 4M) indicate that US combined with BG significantly increases intracellular Ca^2^⁺ levels in normal 4T1 cells. However, calcium influx is significantly inhibited in all groups (even in the US+BG group) in TRPV2‐blocked 4T1 cells, confirming the key role of TRPV2 in Ca^2^⁺ influx (Figure 4N). In TRPV2‐overexpressed cells, BG alone significantly increases intracellular Ca^2^⁺ levels due to the abundant presence of TRPV2 (Figure 4O). Moreover, combined US treatment further significantly enhances intracellular Ca^2^⁺ levels compared to the BG group, confirming the activation effect of US on TRPV2. The fluorescence intensity ratio of TRPV2‐blocked cells to 4T1 cells is defined as the OFF state, while the ratio for TRPV2‐overexpressing cells to 4T1 cells is defined as the ON state. Our results (Figure 4P) indicate that the combined group exhibits a significant effect in the ON state. These results confirmed that US can activate the TRPV2 channels, resulting in an increased influx of Ca^2+^ into breast cancer cells in the presence of BG.

### 2.5 Induction of Mitochondrial Dysfunction and Apoptosis through BG Combined with US Treatment

**Figure 5:**
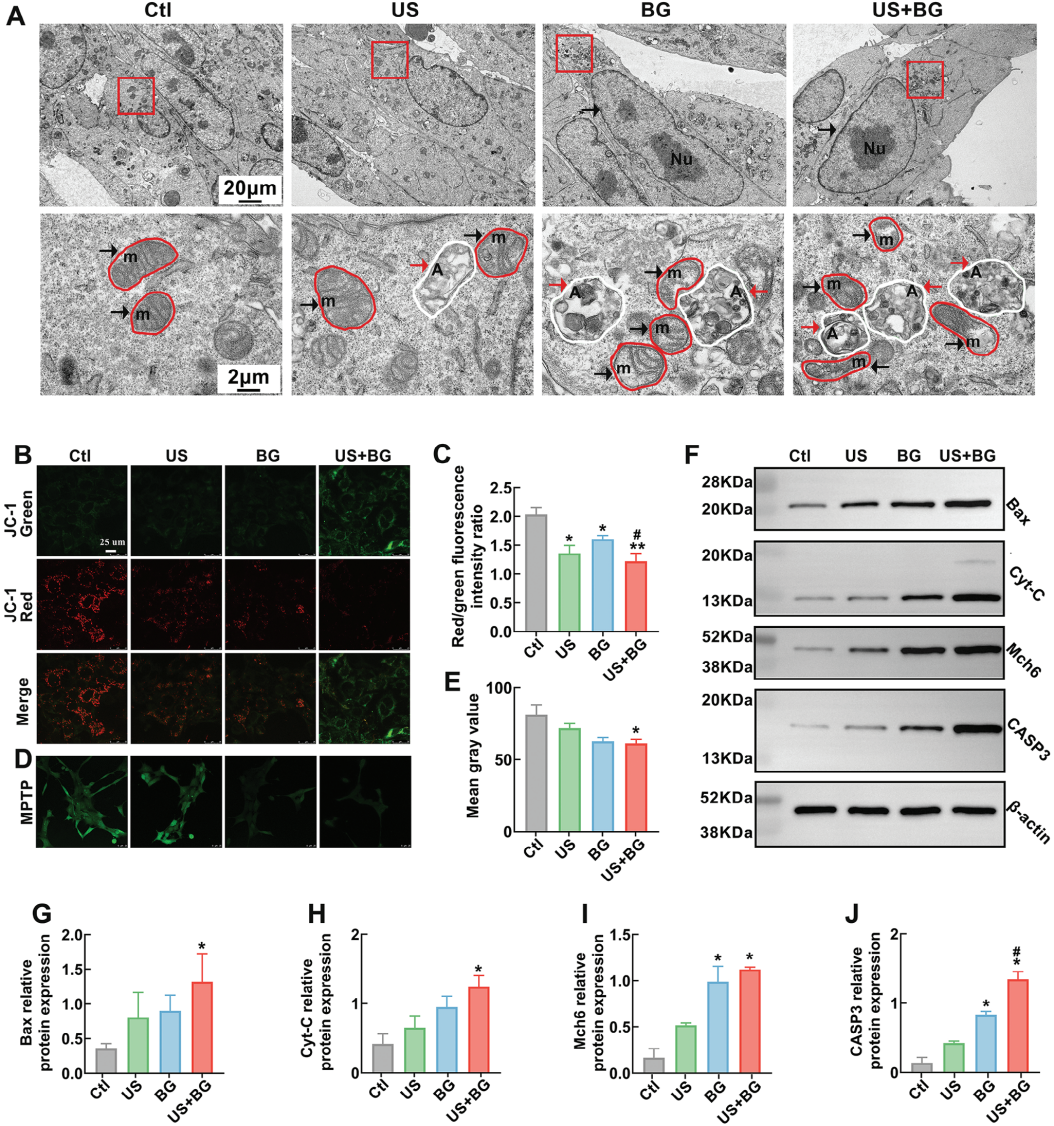
Induction of mitochondrial dysfunction and apoptosis through BG combined with US treatment. A) TEM images of 4T1 cells under various treatments. Nu: the nucleus; m: mitochondria; A: autophagosomes. The red box in the upper images represents the enlarged regions of lower images; the red box in the lower images indicates mitochondria, and the white box indicated autophagosomes. B) The representative images of JC‐1 staining of 4T1 cells in each group. The green fluorescence indicates a reduction in membrane potential, while the red fluorescence indicates a normal mitochondria membrane potential. C) Quantitative analysis of mitochondrial membrane potential. D) Representative fluorescent images for the detection of MPTP opening. The diminished green fluorescence indicates the opening of MPTP. E) Quantitative results of MPTP opening. F) Western blot analysis results of Bax, Cyt‐C, Mch6 (Caspase 9), and CASP3 (Caspase‐3) proteins in 4T1 cells treated with different treatments. Quantitative results of the protein expression levels: G) Bax; H) Cyt‐C; I) Mch6; J) CASP3. Data are presented as mean ± SD (*n* = 3). Statistical significance was determined using one‐way ANOVA followed by Tukey's multiple comparison tests. *: compared with the control group, *p < 0.05, **p < 0.01; #: compared with the BG group, #*p* < 0.05.

Calcium overload typically leads to mitochondrial dysfunction and subsequent cell apoptosis. Therefore, mitochondrial function was systematically analyzed after 4T1 cells were subjected to different interventions. First, transmission electron microscopy (TEM) was used to directly observe the submicroscopic structures of mitochondria (**Figure** **5**A). The mitochondria in the control group and US group exhibited normal morphology with clear cristae. In the BG group, mitochondrial cristae were shortened and loosened, and autophagosomes were observed. While, in US+BG group, the mitochondria showed a more abnormal morphology with obvious disorganized mitochondrial cristae. Furthermore, the mitochondria exhibited signs of condensation and swelling with an increased number of autophagosomes. The TEM imaging results demonstrated that the combination of US and BG could significantly change the morphology of mitochondria in 4T1 cells and increase the formation of autophagosomes.

Decreased mitochondrial potential is a key indicator of mitochondrial dysfunction. To assess this, the mitochondrial membrane potential of cells in each group was analyzed using JC‐1 staining, where red fluorescence indicates normal membrane potential and green fluorescence indicates reduced membrane potential. The results (Figure 5B) showed a significant reduction in mitochondrial membrane potential in the US, BG, and US+BG groups compared to the control group. Statistical analysis revealed the most pronounced reduction in the US+BG group among the treatments (Figure 5C). Next, the opening of the mitochondrial permeability transition pore (MPTP) was measured using the fluorescent probe Calcein‐AM. As shown in Figure 5D,E, the results demonstrated that the combination of US and BG significantly promoted MPTP opening compared to other groups. All these results confirmed that US combined with BG could significantly induce mitochondria dysfunction in 4T1 cells.

Mitochondria dysfunction can lead to ROS accumulation, triggering the mitophagy and cell apoptosis. The ROS detection results (Figure S9, Supporting Information) demonstrated that US combined with BG treatment significantly increased intracellular ROS production. Following this, key proteins involved in mitochondrial autophagy and apoptosis pathways were analyzed by Western blot (Figure 5F and Figure S10 (Supporting Information)). It was found that Bcl‐2 Associated X protein (BAX), Cytochrome c (Cyt‐C), Machado‐Joseph Disease protein 6 (Mch6), and Caspase 3(CASP3) proteins were significantly upregulated in US combined with BG group (Figure 5G–J). These findings suggest that the combination of BG and US induces mitochondrial dysfunction, leading to excessive mitophagy, which ultimately activates apoptotic pathways in 4T1 cells.

### 2.6 The Enhanced Antitumor Effects of BG Combined with US in TRPV2‐Overexpressed Models

**Figure 6:**
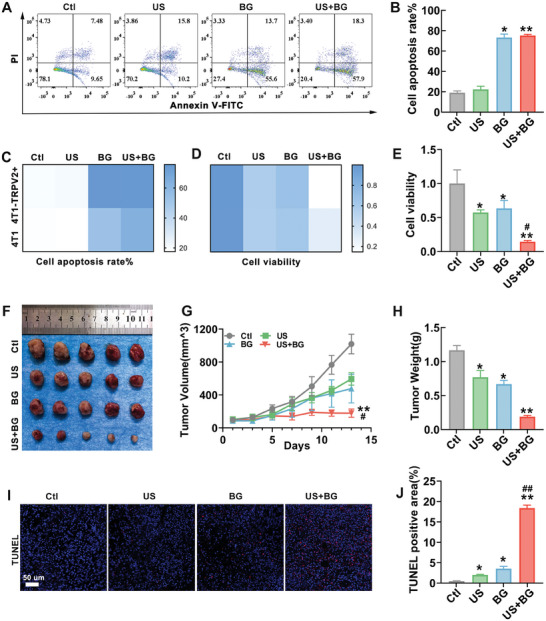
The enhanced antitumor effects of BG combined with US in overexpressed TRPV2 models. A) Annexin V–fluorescein isothiocyanate (FITC)/propidium iodide (PI) staining flow cytometry was used to assess apoptosis rates in TRPV2‐overexpressing 4T1 cells under different treatments, with 0.7 W US for 5 min. B) Quantitative analysis of apoptosis rates. C) Heatmap comparing apoptosis rates between TRPV2‐overexpressing 4T1 cells and nontransfected 4T1 cells, with darker colors indicating higher apoptosis rates. D) Heatmap comparing proliferation rates between TRPV2‐overexpressing and nontransfected 4T1 cells, with lighter colors representing lower proliferation rates. E) CCK‐8 assay measuring the effect of different treatments on the proliferation rates of TRPV2‐overexpressing 4T1 cells. F) Representative images of tumors from an in situ breast cancer model in BALB/c mice using TRPV2‐overexpressing 4T1 cells following various treatments. G) Tumor volume growth curves during treatment. H) Quantitative analysis of tumor weight posttreatment. I) Representative TUNEL fluorescence staining images of tumor tissues. J) Quantitative analysis of TUNEL staining results. Data are presented as mean ± SD (*n* = 5). Statistical significance was determined using one‐way ANOVA followed by Tukey's multiple comparison tests. *: compared with the control group, *p < 0.05, **p < 0.01; #: compared with the BG group, #*p* < 0.05; ##*p* < 0.01.

To further validate the enhanced antitumor efficacy of BG combined with US in TRPV2‐overexpressing breast cancer cells, both in vivo and in vitro experiments were conducted. Cells were subjected to different interventions, and apoptosis was assessed using flow cytometry 24 h later. The results indicated that both BG group and BG combined with US group significantly promoted apoptosis in TRPV2‐overexpressed cells compared to the control group (**Figure** **6**A,B). The combined intervention group exhibited a higher apoptosis rate in TRPV2‐overexpressed cells compared to 4T1 cells (Figure 6C). The impact of various interventions on the proliferation of TRPV2‐overexpressed cells was evaluated using the CCK‐8 assay. The results demonstrated that the combined intervention group had a stronger inhibitory effect on the proliferation of TRPV2‐overexpressed cells (Figure 6D). Compared to the control group, the US, BG, and combined groups significantly inhibited the proliferation of TRPV2‐overexpressed cells, with the combined US treatment showing a markedly enhanced inhibitory effect compared to the BG group (Figure 6E). These results collectively suggest that TRPV2 overexpression in 4T1 cells amplifies the proapoptotic and antiproliferative effects of BG combined US in vitro.

To assess the in vivo antitumor effects of the US combined with BG on TRPV2‐overexpressed breast cancer, 4T1 cells overexpressing TRPV2 were implanted into the third mammary fat pad of mice to establish an orthotopic breast cancer tumor model. The results indicated that the US, BG, and US combined with BG treatment groups all reduced tumor volume, with the combined group showing the most significant effect (Figure 6F). Tumor volume was significantly reduced in the US combined with BG treatment group compared to the BG group (Figure 6G). Measurements of tumor weight revealed a significant reduction in the US combined with BG treatment group (Figure 6H). TUNEL staining of tumor tissues showed that the US, BG, and US combined with BG treatment groups all promoted apoptosis in TRPV2‐overexpressed tumors, with the US combined with BG treatment group showing a significantly enhanced proapoptotic effect compared to the BG group (Figure 6I,J). Additionally, CD4 staining of tumor tissues revealed that the combined treatment significantly increased CD4 expression (Figure S11, Supporting Information), suggesting that the BG combined with US activated the host immune response, leading to increased immune infiltration at the tumor site and enhancing its antitumor effect. Collectively, these results suggest that overexpression of the *TRPV2* gene in 4T1 cells enhances the antitumor efficacy of the combined US and BG treatment. This enhanced effect is likely achieved through US activation of TRPV2 channels, resulting in a significant influx of calcium ions released by BG into the cells, leading to apoptosis.

### 2.7 The Antitumor and Osteogenic Effect of BG Combined with US in the Breast Cancer Bone Metastasis Model

**Figure 7:**
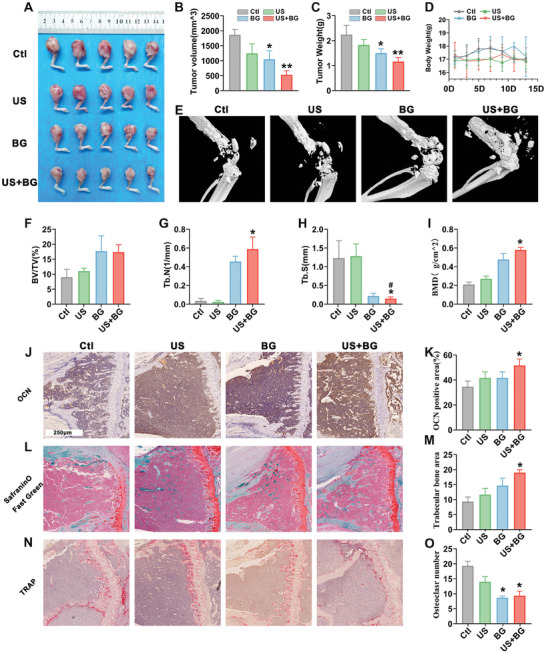
The antitumor and osteogenic effect of BG combined with US in the breast cancer bone metastasis model. A) Representative images of breast cancer bone metastasis model in BALB/c mice following different treatments. B) Quantitative analysis of breast cancer bone metastasis model volume posttreatment. C) Statistical analysis of breast cancer bone metastasis model weight. D) Curve showing the changes in body weight of mice during the treatment period. E) Representative micro‐CT images of breast cancer bone metastasis model after various treatments. F) Statistical analysis of bone volume. G) Quantitative analysis of trabecular number. H) Statistical analysis of trabecular thickness. I) Quantitative analysis of bone mineral density (BMD). J) Representative OCN immunohistochemical staining image and K) quantitative analysis result. L) Representative Safranin O/Fast Green staining image and M) quantitative analysis result. N) Representative TRAP immunohistochemical staining image and O) quantitative analysis result. Data are presented as mean ± SD (*n* = 5). Statistical significance was determined using one‐way ANOVA followed by Tukey's multiple comparison tests. *: compared with the control group, *p < 0.05, **p < 0.01; #: compared with the BG group, #*p* < 0.05.

Based on the previously confirmed antitumor effects of US combined with BG both in vitro and in vivo, we further explored the efficacy of this combination treatment in the breast cancer bone metastasis model. This model was established by implanting 4T1 cells into the right femur of mice, which were then randomly divided into four treatment groups. **Figure** **7**A displays images of breast cancer bone metastasis model volumes in the four groups posttreatment. Statistical analysis indicated that the BG group experienced a reduction in tumor volume, while an effect that was enhanced when combined with US (Figure 7B). This finding was corroborated by measuring the weight of the tumor samples (Figure 7C). No significant differences in body weight were observed among the groups during the treatment period (Figure 7D). To further verify the therapeutic effects, the femurs of the mice were examined using micro‐CT (Figure 7E). The results demonstrated that the combined intervention promoted bone volume growth (Figure 7F), increased trabecular number (Figure 7G), reduced trabecular separation (Figure 7H), and increased bone density (Figure 7I). These findings suggest that US combined with BG not only inhibits tumor growth in breast cancer bone metastasis model but also promotes bone formation.

The expression of the osteogenic gene osteocalcin (OCN) in the implanted site was further examined, revealing that US combined with BG significantly promoted OCN expression (Figure 7J,K). Safranin O–Fast Green Stain also confirmed that the combination treatment significantly enhanced bone formation (Figure 7L,M). Additionally, the expression of Tartrate‐Resistant Acid Phosphatase Staining (TRAP) was assessed, showing that BG inhibited osteoclast activity, with this effect being enhanced by US (Figure 7N,O). In summary, these results demonstrate that the combination of US and BG not only inhibits the growth of the breast cancer bone metastasis model but also promotes osteogenesis.

## 3 Discussion

Breast cancer frequently metastasizes to bone, severely impacting patient survival and quality of life. Advances in diagnostics and treatments have increased surgical interventions for breast cancer with bone metastasis.^[^ ^14^ ^]^ However, surgery often results in large tissue defects, raising the risk of residual tumor recurrence, which complicates wound healing. Additionally, antitumor therapies often conflict with tissue repair, as both have opposing requirements. For example, antiangiogenic therapies suppress tumor growth by inhibiting blood vessel formation but also impair necessary vascularization for tissue repair. Similarly, chemotherapy's broad cytotoxicity indiscriminately affects healthy osteoblasts and endothelial cells, hampering tissue recovery and reducing biocompatibility. Thus, selective targeting of cancer cells while preserving normal tissue remains a critical goal for effective therapy.

TRPV2 is a key marker for tumor invasion and progression,^[^ ^10a^ ^]^ with elevated expression in tumor cells compared to normal stem cells and osteoblasts (Figure S12, Supporting Information). TRPV2‐mediated calcium regulation is required for tumor growth and metastasis, with calcium imbalance associated with invasion, metastasis, and drug resistance.^[^ ^15^ ^]^ Targeting calcium homeostasis may provide beneficial cancer treatments. Therapeutic strategies encompass enhanced calcium influx to activate signaling pathways or calcium‐based nanomaterials for cellular calcium overload, triggering tumor calcification and immune responses through pyroptosis.^[^ ^4^, ^5^ ^]^ However, conventional TRPV2 activators (e.g., 2‐APB, probenecid, CBD) face low specificity and potential side effects.^[^ ^11^, ^16^ ^]^ Thus, identifying a specific and effective method to activate TRPV2 remains challenging and a substantial research focus. In this study, we demonstrated that US effectively controlled the opening of the TRPV2 channel. US, already FDA‐approved for certain localized treatments, offers deep tissue penetration and precise targeting, overcoming the high cell density and pressure gradients in tumors that hinder drug delivery. We observed that the inhibition rate of tumor cells increased progressively with the increase of US intensity and exposure time (Figure S3, Supporting Information).

Simply opening the TRPV2 channel itself is not sufficient to induce the desired therapeutic effects, as it alone does not lead to a significant increase in intracellular calcium ion concentration in tumor cells. In other words, activating TRPV2 alone is not enough to cause calcium overload; an additional calcium source is required to achieve this goal. This is where BG demonstrates its unique advantages. Since the early 21st century, BG is valued in medicine for its bioactivity and repair‐promoting properties.^[^ ^17^ ^]^ Advances in BG fabrication have enabled more controlled morphologies, enhancing its clinical applications.^[^ ^18^ ^]^ Beyond its excellent tissue repair properties, BG offers exceptional biocompatibility, osteoinductivity, and a large surface area, contributing to its anti‐inflammatory and antibacterial effects, useful for clinical repair.^[^ ^19^ ^]^ Considering that the long‐term effects of BG on surrounding tissues and its potential toxicity are critical factors for its clinical application, several studies have explored this aspect and provided valuable insights. For instance, Delpino et al. evaluated the biocompatibility and safety of BG in both in vitro and in vivo models, finding that locally applied BG particles exhibit minimal cytotoxicity and are well‐tolerated by surrounding tissues.^[^ ^20^ ^]^ Rahaman et al. demonstrated that BG can promote tissue regeneration and has no significant adverse effects on the local environment over a 12‐month observation period.^[^ ^21^ ^]^ These findings suggest that BG is highly safe for long‐term therapeutic use. More importantly, BG can rapidly degrade in vivo, releasing calcium, boron, and magnesium ions. These ions not only help create a favorable alkaline environment that promotes osteogenesis and neutralizes the tumor microenvironment but also provide a crucial source of calcium that is essential for achieving calcium overload (Figure S13, Supporting Information).^[^ ^22^ ^]^ When we combined US with BG, the antitumor efficacy was further enhanced. US not only effectively opens the TRPV2 channel but also accelerates the release of calcium ions from BG and BG dispersion. This dual action enables tumor cells to take up more calcium ions when the TRPV2 channel is open, leading to calcium overload within the cells. The antitumor effect is amplified when TRPV2 is overexpressed and diminished when TRPV2 is inhibited (Figure 4L). This strategy promotes calcium overload selectively in tumor cells, providing a controlled, potent approach for cancer therapy. Furthermore, combining US with BG not only targets tumors but also enhances immune responses, as indicated by increased CD4 expression (Figure S11, Supporting Information) aligning with recent findings from Yang et al. in 2022, on immune stimulation in cancer therapy.^[^ ^13^, ^23^ ^]^

**Figure 8:**
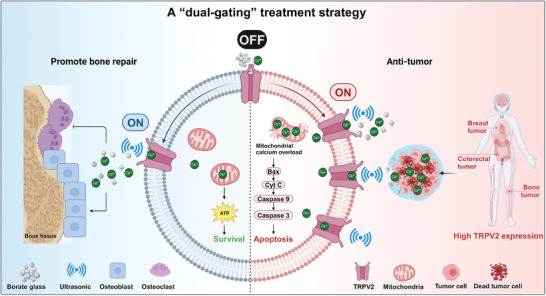
Graphical summary of the proposed mechanism. The dual‐gating strategy, combining ultrasound activation of TRPV2 channels and borate‐glass‐induced calcium overload, innovatively triggers mitochondrial autophagy to induce tumor cell apoptosis, while also promoting bone tissue regeneration in bone metastasis models (the figure was created with BioRender.com).

The combination of US and BG exhibits antitumor activity and osteoprotective effects via two distinct mechanisms. First, low‐frequency US activates TRPV2, which remains “OFF” with BG alone. US triggers TRPV2 to be “ON,” triggering substantial calcium influx from BG and inducing apoptosis. Second, US facilitates controlled calcium ion release, leading to tumor cell autophagy‐related apoptosis. By contrast, normal osteoblasts, with lower TRPV2 expression, absorb fewer calcium ions, preserving bone balance. Our results suggest that US‐enhanced BG effectively inhibits tumor growth while promoting bone regeneration, presenting a promising, targeted strategy for cancer treatment (**Figure** **8**).

## 4 Conclusion

This study presents an innovative, effective, and highly specific antitumor strategy that combines US with BG in a dual‐gating approach for breast cancer treatment. US stimulation not only promotes ion release from BG but also activates the TRPV2 channel, disrupting calcium homeostasis in breast cancer cells, inducing mitochondrial autophagy, and ultimately triggering apoptosis. Due to the differential expression of TRPV2 between tumor cells and normal cells, this strategy significantly enhances therapeutic specificity while sparing healthy tissue. In both subcutaneous and orthotopic breast cancer mouse models, the US–BG combination exhibited strong antitumor effects, inducing apoptosis with good tolerability. Remarkably, in breast cancer bone tumor models, this therapy not only inhibited tumor growth but also promoted osteogenesis. These promising results suggest that this strategy may be applicable to other tumors with high TRPV2 expression. Future research should explore the effects of varying US frequencies and intensities on TRPV2 activation, as well as refine BG composition and structure to optimize ion release for more precise therapeutic outcomes, broadening its potential in cancer treatment.

## 5 Experimental Section

### Synthesis and Characterization of Borate Glass

The micrometer‐sized BG was synthesized by melting and quenching technique based on the previously reported method.^[^ ^24^ ^]^ Briefly, the required amounts of H_3_BO_3_ (58.237 g), CaCO_3_ (18.654 g), Na_2_CO_3_ (3.59 g), 4MgCO_3_·Mg(OH)_2_·5H_2_O (6.58 g), K_2_CO_3_ (9.362 g), and NaH_2_PO_4_·2H_2_O (5.284 g) were uniformly mixed using a ternary mixer and then added to a platinum crucible. The mixture was placed in a muffle furnace and melted at 1200 °C for 60–120 min. Afterward, the molten glass was poured into an ice‐water bath to cool and solidify to get the raw glass powder. Finally, the raw powder was crushed, ground, and sieved through an antioxidant steel mesh to obtain borate bioactive glass.

The morphology and compositions of BG were characterized by a SEM equipped with an energy‐dispersive spectroscopy (Carl Zeiss, Germany) at accelerating voltage of 20 kV. XRD analysis was performed for crystallographic characterization in the 2*θ* range of 10°–80°. The size distribution was examined by a Zetasizer system (Mastersizer 3000, UK). And coupled plasma spectrometer (ICP, Vista AX, ThermoFisher) was used to analyze the ion release.

### Cell Culture and Transfection

4T1 breast cancer cells (ZQ0201) and mouse BMSC (ZQ0465) were purchased from the Zhongqiao Xinzhou Science and Technology Co. (Shanghai, China). 4T1 cells were cultured in 1640 complete medium and mouse BMSCs were cultured in α‐MEM complete medium, both of which were supplemented with 10% serum and 1% penicillin and streptomycin (Antibiotic‐Antimycotic; Gibco, USA) in a 37 °C incubator with a humidified atmosphere of 5% CO_2_. Unless mentioned elsewhere, cells were incubated with BG (1 mg mL^−1^) for 6 h and were then treated with US for 5 min (1 MHz, 0.7 W, 50% duty cycle). Subsequent analyses were performed 24 h later. To establish the stable cells, 4T1 cells were infected with lentiviral scramble and the cells were selected by 2ug mL^−1^ puromycin for 48–72 h, or until all nontransfected control cells were eliminated. Lentivirus and puromycin were purchased from Obio Technology (Shanghai, China). For experiments with ruthenium red blockage, cells were incubated with 20 µm ruthenium red (Sigma‐Aldrich) for 1 h.

### Cell Viability and Apoptosis Assay

Cell viability was assayed by CCK‐8 (Beyotime Biotechnology, Shanghai, China) according to the manufacturer's instructions. Cells were seeded in 96‐well plates at 5000 cells per well and were allowed to adhere overnight. The cells were then exposed to BG and US for 24 h. Then, CCK‐8 solution was added at 37 °C. 1 h later, absorbance at 450 nm was measured with a microplate reader (Thermo Fisher Scientific).

For apoptosis assay, cells were seeded and incubated in 12‐well plates at a concentration of 100 000 cells per well. 24 h later, the cells were exposed to BG and US treatment. Another 24 h later, cells were stained with Annexin V–fluorescein isothiocyanate (FITC)/propidium iodide (PI) staining tool kit (Beyotime Biotechnology, Shanghai, China) according to the manufacturer's instruction, and apoptosis was detected by flow cytometry (BD Biosciences).

### Live/Dead Staining

The Calcein‐AM/PI living/dead cell double staining kit (Dojindo Laboratories, Shanghai, China) was used for a living and dead cell level analysis. Cells were incubated overnight in 12‐well plates at a density of 100 000 cells per well. Then, the cells were exposed to BG and US conditions. 24 h later, the cells were collected and stained according to the instructions. Cell fluorescence was visualized with laser confocal microscope (Leica, Germany). Calcein‐AM was used as the live stain, with live cells appearing green, while PI was used as the dead stain, with dead cells being stained red.

### Establishment of Subcutaneous and Orthotopic Breast Cancer Model

The procedure was approved by the Ethics Committee of Shanghai Tongren Hospital (A2023‐010‐01). Female BALB/C mice (Vital River Laboratory Animal Technology, Shanghai, China), aged 6–8 weeks, were used to establish the subcutaneous breast cancer tumor model. Each mouse was subcutaneously injected with 1 million 4T1 tumor cells. Once the tumor volume reached ≈100 mm^3^, the mice were randomly divided into six groups (*n* = 6), namely Control group (without intervention), US group (treated with only US), BG1 (treated with 1 mg BG) group, BG group (treated with 5 mg BG), US+BG1 group (treated with 1 mg BG and US), and US+BG group (treated with 5 mg BG and US). The ultrasound (0.7 W, 1 MHz) was applied to the tumor sites for 20 min in each ultrasound‐treated group. The treatments were implemented every other day with total six treatments. 15 days after the first treatment, mice were anesthetized and euthanized to collect the subcutaneous tumors for further analysis. The tumor samples in each group were subsequently used for histological examination.

Female BALB/C mice, aged 6–8 weeks, were used to establish an orthotopic tumor model. Each mouse was injected with 1 million 4T1 tumor cells into the fourth mammary fat pad. Once the tumor volume reached ≈100 mm^3^, the mice were randomly divided into four groups (*n* = 5): Control group (without intervention), US group (treated with US only), BG group (treated with 5 mg BG), and US+BG group (treated with 5 mg BG and US). The ultrasound (0.7 W, 1 MHz) was applied to the tumor sites for 20 min in each ultrasound treated group. Treatments were administered every other day for a total of six sessions. Tumor volume and mouse body weight were measured daily throughout the treatment period. 15 days after first treatment, mice were anesthetized and euthanized to collect the orthotopic tumors for further analysis. The tumor samples in each group were subsequently used for histological examination and immunohistochemical analysis.

### Histopathology and Immunohistochemistry Analysis

Tumor tissues were excised from treated tumor‐bearing mice and fixed in 4% paraformaldehyde (absin, Shanghai) after treatment. Paraffin sections were prepared and sliced into 5 µm sections. The sections were subjected to H&E staining (Servicebio, China). For Ki67 staining, tumor sections were incubated with anti‐Ki67 antibody (1:500, Servicebio, China). The sections were washed twice with DPBS and then incubated with horseradish‐peroxidase (HRP)‐conjugated secondary antibody for 1 h (1:3000, Servicebio, China). Images were captured using a Nikon DS‐U3 microscope (Nikon, Japan). Apoptosis induction in tumor tissues was evaluated using the TUNEL assay, and nuclei were stained with DAPI. TUNEL‐positive signals were detected using a Nikon Eclipse C1 fluorescence microscope (Nikon, Japan). For CD4 staining, tissues were incubated overnight at 4 °C with rabbit anti‐CD4 antibody (1:400, Servicebio, China). The next day, the tissues were rinsed and incubated with Cy3‐conjugated anti‐rabbit IgG (1:800, Servicebio, China) for 1 h, after which the sections were examined under a microscope.

### Mitochondria Function Analysis

Mitochondrial membrane potential measurement was monitored using the JC1‐Mitochondrial membrane potential assay kit (Beyotime, Shanghai, China). 4T1 cells were seeded into confocal dishes and incubated for 24 h (37 °C, 5% CO_2_). Subsequently, four different interventions were applied (Control; US; BG; US+BG). Then, 1 mL of JC‐1 staining solution was added to each confocal dish, mixed, and incubated at 37 °C for 20 min. The supernatant was then removed, and cells were washed twice with JC‐1 staining buffer (1×). Finally, a laser confocal microscope was used to detect the mitochondrial membrane potential (Leica, Germany).

The sensitivity of MPTP opening to Ca^2+^ was assessed by MPTP Assay Kit (Beyotime, Shanghai, China), according to the manufacturer's protocol. Briefly, 4T1 cells were washed twice with phosphate‐buffered saline (PBS), loaded with Calcein‐AM at 37 °C for 15 min, then incubated with 1 mm CoCl_2_ for 1 h. After washing with PBS, the cells were imaged under a confocal microscope using a 488 nm argon laser and a 525 nm emission filter (Leica, Germany).

### TEM Observation

Cells were divided into four groups: Control, US (5 min ultrasound), BG (1 mg mL^−1^), and US+BG (5 min ultrasound + 1 mg mL^−1^ BG). Each group received the designated intervention for 24 h. Then, cells were collected by trypsinization and centrifugation at 1000 rpm for 5 min. Cell pellets were washed twice with PBS. Cells were fixed in 2.5% glutaraldehyde in 0.1 m cacodylate buffer (pH 7.4) for 2 h at 4 °C. Postfixation was performed with 1% osmium tetroxide in 0.1 m cacodylate buffer for 1 h at room temperature. Samples were dehydrated by graded ethanol (50%, 70%, 90%, and 100%). Further dehydration was done with propylene oxide for 10 min. Samples were infiltrated with a mixture of propylene oxide and epoxy resin (1:1) for 1 h, followed by pure epoxy resin overnight. Samples were embedded in fresh epoxy resin and polymerized at 60 °C for 48 h. Ultrathin sections (70–90 nm) were cut using an ultramicrotome. Sections were collected on copper grids and stained with uranyl acetate and lead citrate for contrast. Sections were examined using a TEM at an accelerating voltage of 80–100 kV. Images were captured digitally for analysis.

### Transcriptome Sequencing of Subcutaneous Tumors

The subcutaneous tumor samples (*n* = 3) of control and US+BG group (treated with 5 mg BG+US) were used for transcriptome sequencing. Tumors were excised from the animals under sterile conditions and immediately snap‐frozen in liquid nitrogen. Total RNA was extracted using Trizol reagent. RNA purity and quantification were assessed using a NanoDrop 2000 spectrophotometer (Thermo Scientific, USA), and RNA integrity was evaluated using an Agilent 2100 Bioanalyzer (Agilent Technologies, Santa Clara, CA, USA). Samples that passed quality control were used for subsequent library construction. Ribosomal RNA was removed using the Ribo‐off rRNA Depletion Kit (Vazyme, Nanjing, China), and the transcriptome library was constructed using the VAHTS Universal V6 RNA‐seq Library Prep Kit according to the manufacturer's instructions. Whole transcriptome sequencing and analysis were performed by Shanghai OE Biotech Co., Ltd. (Shanghai, China). Differential expression gene analysis was conducted using the DESeq2 software. Subsequently, KEGG pathway enrichment analysis of differentially expressed genes was performed based on the hypergeometric distribution algorithm to identify significantly enriched functional terms. GSEA was performed using predefined gene sets, where genes were ranked according to their differential expression between the two types of samples, and then tested for enrichment at the top or bottom of the ranked list.

### Detection of Calcium Ions in Cell Supernatant

The supernatants from cells subjected to different treatments were collected, and the ion concentration in the cell supernatants was measured using an ICP spectrometer (Vista AX, ThermoFisher).

### Western Blot Analysis

4T1 cells were seeded in a 12‐well plate at a density of 100 000 cells per well. The cells were allowed to adhere and grow overnight under standard culture conditions (37 °C, 5% CO_2_). The next day, the cells were subjected to US and BG interventions. After 24 h of treatment, the cells were gently washed with cold PBS and harvested by scraping them into RIPA buffer containing protease inhibitors. The cell lysates were incubated on ice for 30 min, with occasional vortexing, and then centrifuged at 12 000 *g* for 15 min at 4 °C to remove cell debris. The supernatant containing total protein was collected, and the protein concentration was quantified using a BCA protein assay kit. The separated proteins were transferred to a PVDF membrane using a wet transfer system. The membrane was blocked with 5% skim milk in TBST for 1 h at room temperature. The primary antibodies, diluted in TBST containing 5% skim milk, were then incubated with the membrane overnight at 4 °C. The primary antibodies used were as follows: Bcl‐2 (GB154380, Servicebio), BAX (GB114122, Servicebio), Cytc (GB11080, Servicebio), caspase (GB11053, Servicebio), c‐caspase3 (ab32042, Abcam), parkin (GB113802, Servicebio), LC3 (GB113801, Servicebio). Anti‐β‐actin (GB15003, Servicebio) was used as a loading control. After incubation with the primary antibodies, the membrane was washed 3 times with TBST and then incubated with HRP‐conjugated goat anti‐rabbit secondary antibody (GB23303, Servicebio) for 1 h at room temperature. The membrane was washed 3 more times with TBST. Protein bands were visualized using an enhanced chemiluminescence detection kit and captured with a chemiluminescence imaging system (Bio‐Rad). Band intensity was quantified using ImageJ software.

### Intracellular Calcium Analysis

4T1 cells were seeded in confocal dishes at a density of 100 000 cells per dish. The cells were allowed to adhere and grow overnight under standard culture conditions (37 °C, 5% CO₂). After 24 h, the cells underwent the required experimental treatment. Following treatment, the cells were stained using the Flow‐4 calcium assay kit (Servicebio, China) according to the manufacturer's instructions. Briefly, the staining solution was prepared and added to the cells. The cells were incubated with the staining solution at 37 °C for 30 min to allow the dye to enter the cells. After staining, the cells were gently washed with warm PBS to remove excess dye. The dish was then placed on the stage of a confocal microscope. Intracellular calcium levels were detected, and images were captured using the 494 nm excitation and 528 nm emission settings for Flow‐4 dye. Image analysis was performed using ImageJ software to quantify the fluorescence intensity, which corresponded to the intracellular calcium levels.

### Establishment of Breast Cancer Model of Bone

The breast cancer model at bone site was established according to previously reported method.^[^ ^25^ ^]^ Briefly, female BALB/C mice aged 6–8 weeks were anesthetized, and 1 million 4T1 breast cancer cells were injected into the right femur of each mouse using a microsyringe. 4 days after cell injection, the mice were randomly divided into four groups (*n* = 6): Control group (without intervention), US group (treated with US only), BG group (treated with 30 mg BG), and US+BG group (treated with 30 mg BG and US). A surgical procedure was performed under aseptic conditions. A small incision was made over the femur, and a 1 mm diameter hole was drilled vertically into the femur using a dental drill, and solid powder of BG (30 mg per animal) was then implanted into the drilled defects in the mice of BG group and US+BG group. Following the implantation, the skin incisions in each mouse were sutured and disinfected. The mice in US group and US+BG group were treated with US (0.7 W, 50 MHz, 20 min) every other day for a total of six sessions. Throughout the treatment period, the body weight of mouse was measured daily. 15 days after BG implantation, mice were anesthetized and euthanized, and the complete femurs were collected for gross view examination, micro‐CT scanning, and immunohistochemical analysis.

### Microcomputed Tomography

The femoral samples from the breast cancer bone metastasis model after treatment were collected and examined using a micro‐CT system (vivaCT 80, SCANCO Medical AG) to assess bone repair. Briefly, undecalcified samples were scanned at a resolution of 18 µm. After 3D reconstruction, new bone formation was evaluated using indices such as bone mineral density (BMD) and bone volume fraction in the defect area.

### Immunohistochemistry Staining of Bone Tissue

The femoral bone tissues of treated mice were excised and fixed in 4% paraformaldehyde (absin, Shanghai). Paraffin sections were dewaxed to water and then subjected to TRAP staining (Servicebio, China). The sections were circled with a hydrophobic barrier using a pap pen and placed in a humid box, then incubated with distilled water at 37 °C for 2 h. After discarding the distilled water, the sections were placed back in the humid box, and freshly prepared filtered TRAP incubation solution was added. The sections were then incubated at 37 °C for 20 min. Hematoxylin solution was added for nuclear counterstaining for 15 s. The sections were then dehydrated for examination under a microscope. For Safranin–Fast Green staining of bone tissue paraffin sections, the paraffin sections were dewaxed to water and stained using the Safranin–Fast Green staining kit (Servicebio, China). The sections were placed in the Fast Green staining solution for bone tissue for 1–5 min, then washed with water to remove excess stain until the cartilage appeared colorless. The sections were briefly dipped in 1% hydrochloric acid alcohol for 10 s and rinsed with tap water. The sections were then placed in the Safranin staining solution for bone tissue for 1–5 s, rapidly dehydrated with absolute ethanol, and examined under a microscope. For OCN immunohistochemistry analysis, each specimen was first decalcified for ≈2 weeks, then dehydrated through a graded alcohol series, embedded in paraffin, and cut into 5 µm sections. OCN staining was then performed (Servicebio, China) to assess osteogenesis in the specimens.

### Statistical Analysis

GraphPad Prism 8 software was applied for statistical analysis. Quantitative data were expressed as mean ± standard deviation (SD). Statistically significant differences (*p*) were analyzed by *t*‐test, one‐way ANOVA, and Tukey's multiple comparison tests. A value of *p* < 0.05 was considered statically significant and was represented by the symbol “*” “#,” a value of *p* < 0.01 was represented by “**” “##.”

## Conflict of Interest

The authors declare no conflict of interest.

## Author Contributions

H.Z. and L.O. contributed equally to this work. H.Z.: methodology, conducted animal experiments, formal analysis, visualization, original paper writing, and revision; L.O.: paper writing, revision, and funding acquisition; Y.H.: conducted animal experiments and formal analysis; J.T.: physical and chemical characterization of materials; C.L.: design and synthesis of materials, formal analysis; Q.W., R.H.: formal analysis; X.L., H.P., Y.L.: conceptualization, methodology, project administration, supervision, funding acquisition, resources.

## Supporting information



Supporting Information

## Data Availability

The data that support the findings of this study are available from the corresponding author upon reasonable request.
