# Supplementary material for: Dual‐Gating Strategy: Ultrasound Activation of TRPV2 Channels and Borate‐Glass‐Induced Calcium Overload for Tumor Suppression
Source: Adv Sci (Weinh). 2025 Feb 27;12(16):2414676. doi: 10.1002/advs.202414676 (PMC12021067; doi:10.1002/advs.202414676)
Supplement: Supplementary file 1 — Supporting Information [file ADVS-12-2414676-s001.docx]

Supporting information

**Dual-Gating Strategy: Ultrasound Activation of TRPV2 Channels and Borate Glass-Induced Calcium Overload for Tumor Suppression**

*Haihong Zhu^1,2^, Liping Ouyang^2,3^, Yangguang Huang^2^, Ji Tan^4^, Chunyu Liu^5^, Qian Wang^2^, Rongkun Huang^2^, Wing Tak Wong^6^, Xuanyong Liu^4,7,*^, Haobo Pan^5,*^, Yun Liao^1,2,*^*

^1^ Department of Pharmacy, Shanghai General Hospital, Shanghai Jiao Tong University School of Medicine, Shanghai 200080, China

^2^ Department of Pharmacy, Tongren Hospital, Shanghai Jiao Tong University School of Medicine, Shanghai 200336, China

^3^ Hongqiao International Institute of Medicine, Shanghai Jiao Tong University School of Medicine, Shanghai, 200336, China

^4^ State Key Laboratory of Advanced Ceramics , Shanghai Institute of Ceramics, Chinese Academy of Sciences, Shanghai 200050，China

^5^ Shenzhen Key Laboratory of Marine Biomedical Materials, CAS-HK Joint Lab of Biomaterials, The Key Laboratory of Biomedical Imaging Science and System，Shenzhen Institute of Advanced Technology, Chinese Academy of Sciences, Shenzhen,518055, China

^6^ School of Life Sciences, Faculty of Science, The Chinese University of Hong Kong, Hong Kong 999077, China

^7^ School of Chemistry and Materials Science, Hangzhou Institute for Advanced Study, University of Chinese Academy of Sciences, Sub-Lane Xiangshan, Hangzhou, 310024, China

1 These authors equally contributed to this work.

1. mail addresses:
   (Xuanyong Liu) [xyliu@mail.sic.ac.cn](mailto:xyliu@mail.sic.ac.cn(X.Liu),)
   (Haobo Pan) hb.pan@siat.ac.cn
   (Yun Liao) [libra_ly@shsmu.edu.cn](mailto:libra_ly@shsmu.edu.cn)

This file includes:

Supplementary Materials: Figure S1 to S14; Table 1

Supplementary Methods


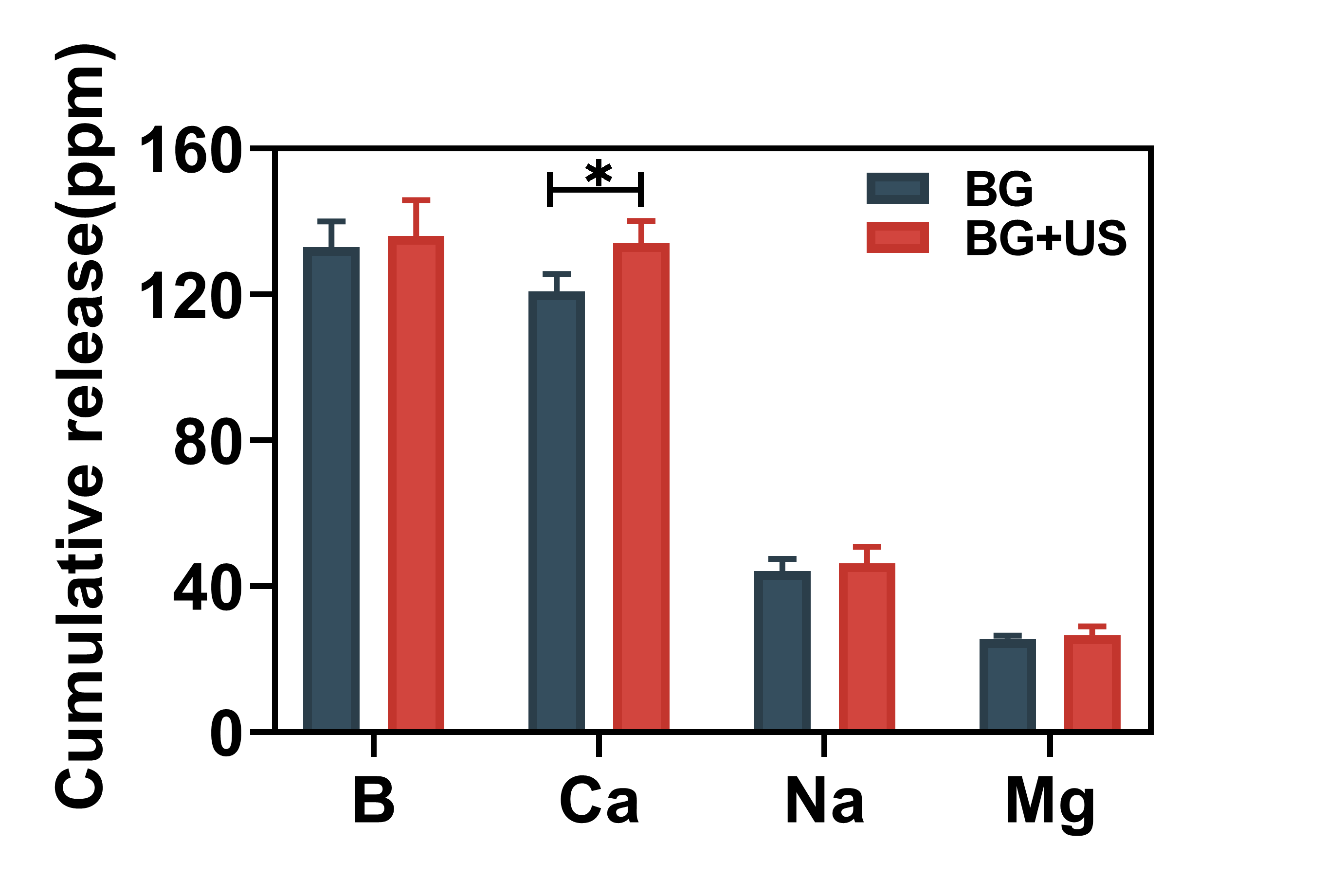


**Figure S1.** The cumulative ion release of borate glass with or without ultrasonic treatment in deionized water. Data are presented as mean ± SD (n = 3). Statistical significance was determined using a t-test. *p < 0.05.


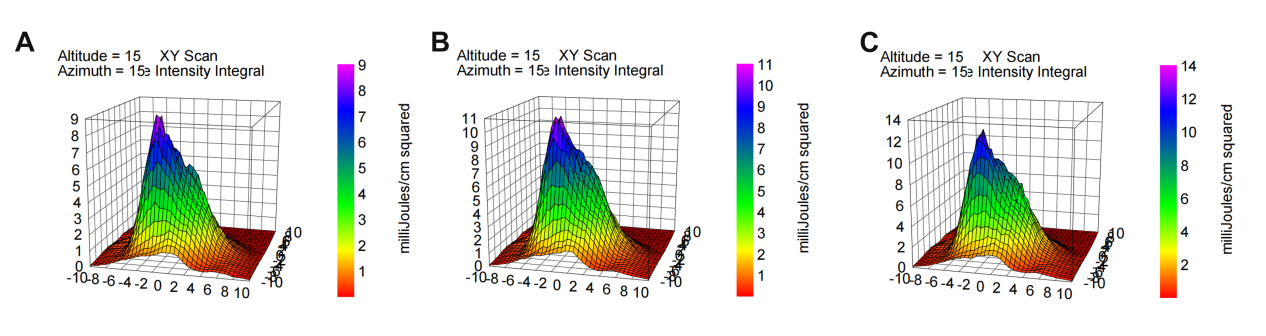


**Figure S2.** Ultrasonic sound field images at (A) 0.2 W/cm², (B) 0.5 W/cm², and (C) 0.7 W/cm².


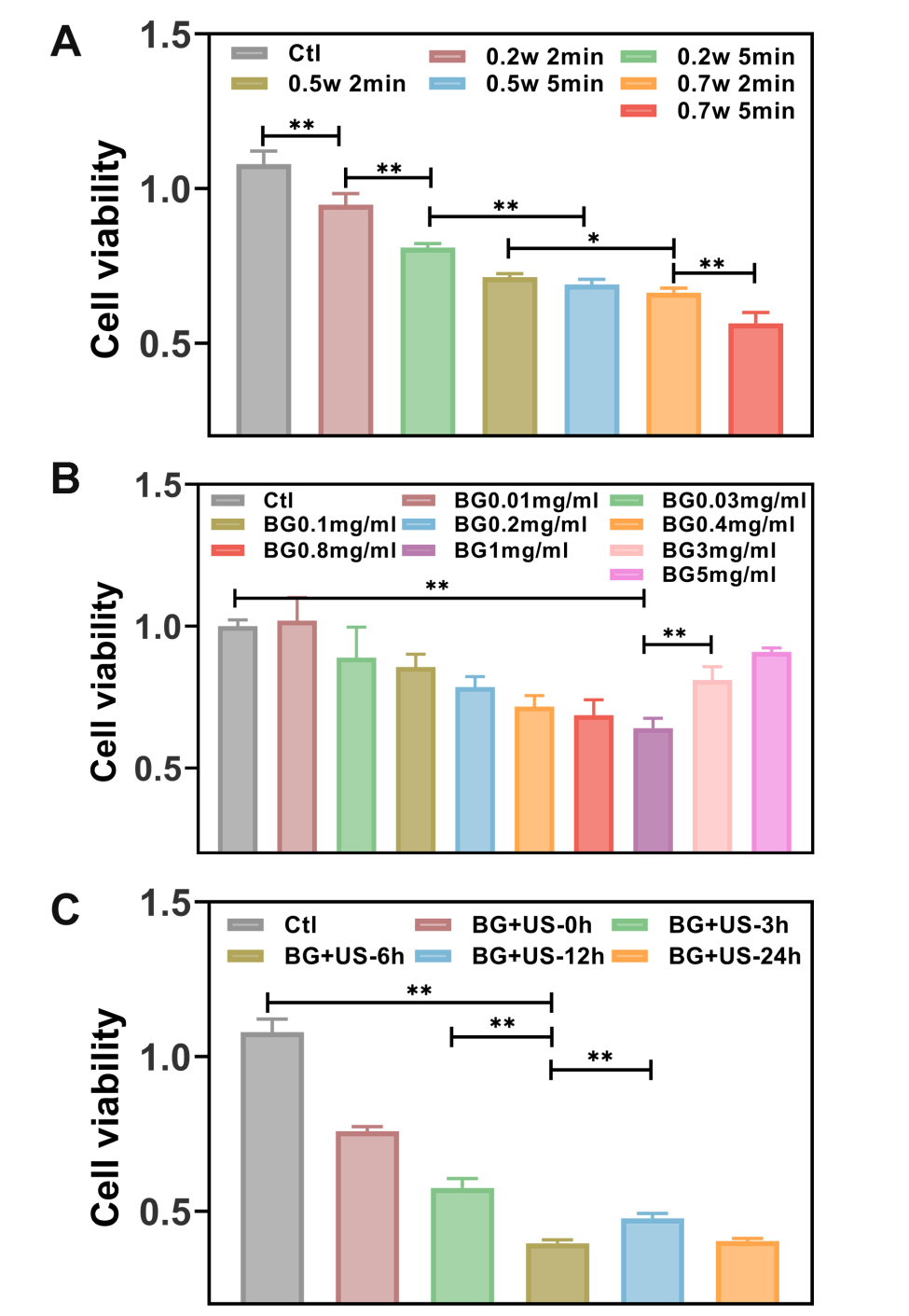


**Figure S3.** (A) Effects of different US intensities and durations on the proliferation rate of breast cancer cells; (B) Effects of different BG concentrations on the proliferation rate of breast cancer cells; (C) Effects of US interventions at various intervals post-incubation with BG on the proliferation rate of breast cancer cells. Data were presented as mean ± SD (n = 6). Statistical significance was assessed by one-way ANOVA followed by Tukey’s multiple comparison test. *p < 0.05, **p < 0.01.


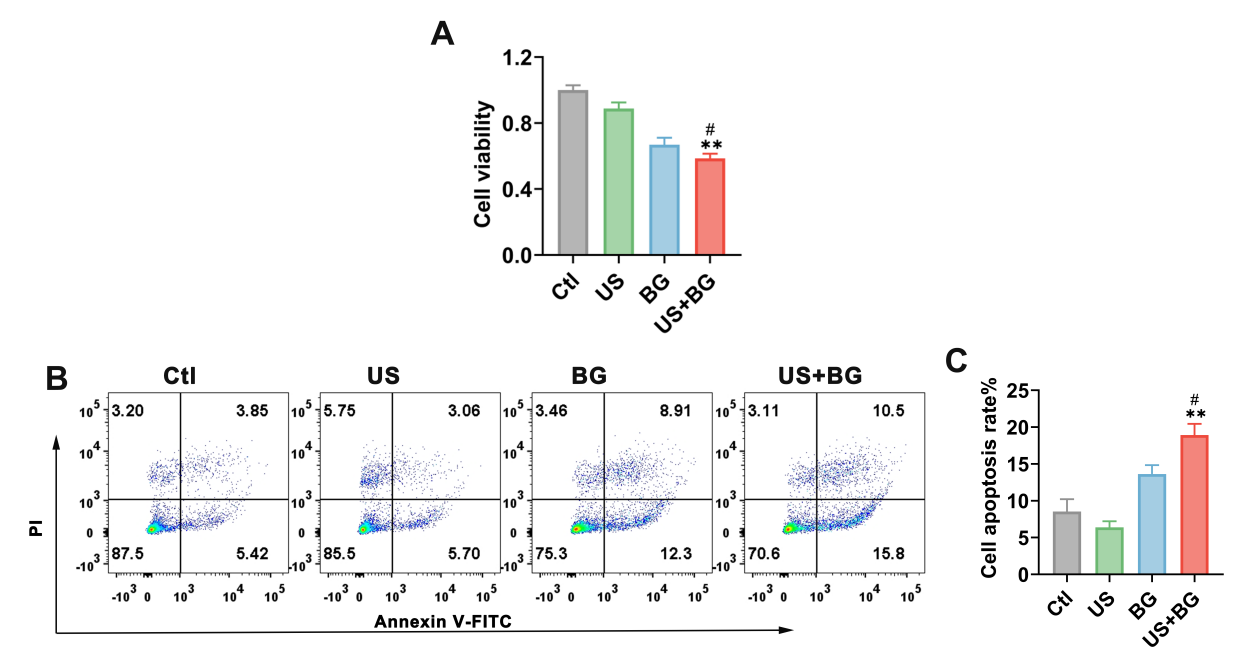


**Figure S4.** Effects of different interventions on the proliferation and apoptosis of MDA-MB-231 cells. (A) Proliferation rate of MDA-MB-231 cells. Apoptosis rate (B) and statistical chart (C) of MDA-MB-231 cells. Data were presented as mean ± SD (n = 3). Statistical significance was assessed by one-way ANOVA followed by Tukey’s multiple comparison test. *: Compared with the control group, *p < 0.05, **p < 0.01; #: Compared with the BG group, #p < 0.05.


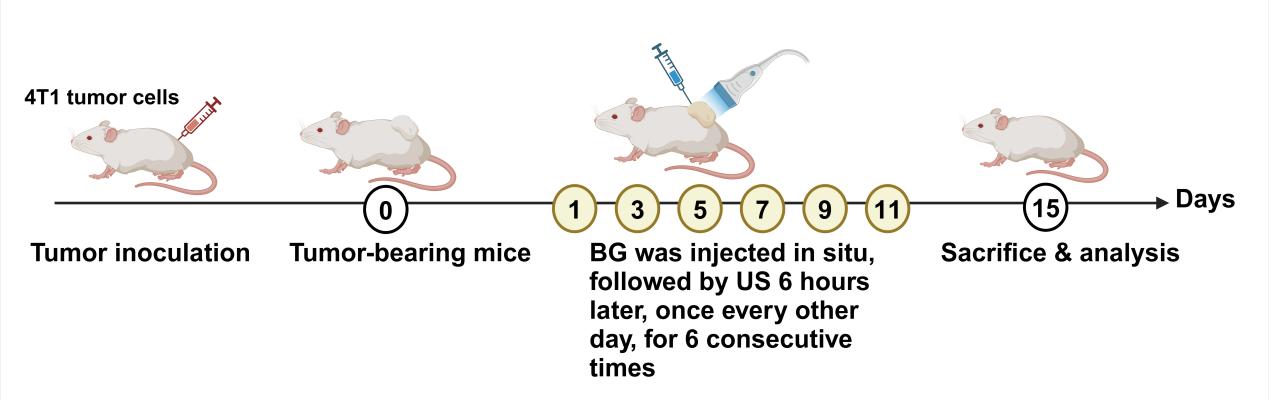


**Figure S5.** Schematic diagram of the establishment and treatment of a subcutaneous breast cancer animal model.


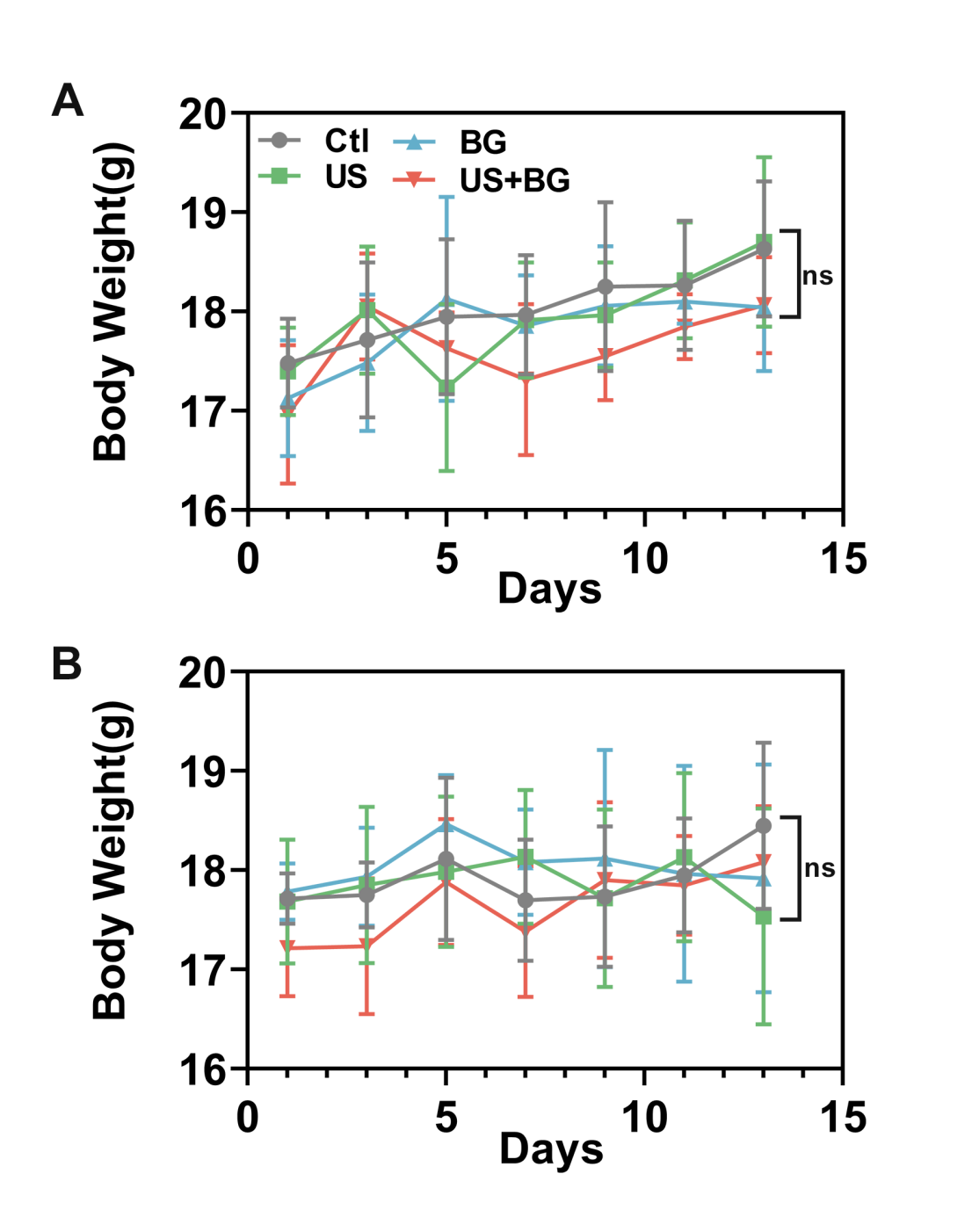


**Figure S6.** (A) Body weight variation curves during the treatment period in a subcutaneous breast cancer animal model; (B) Body weight variation curves during the treatment period in an orthotopic breast cancer animal model. Data were presented as mean ± SD (n = 6). Statistical significance was assessed by one-way ANOVA followed by Tukey’s multiple comparison test. ns means no statistical difference.


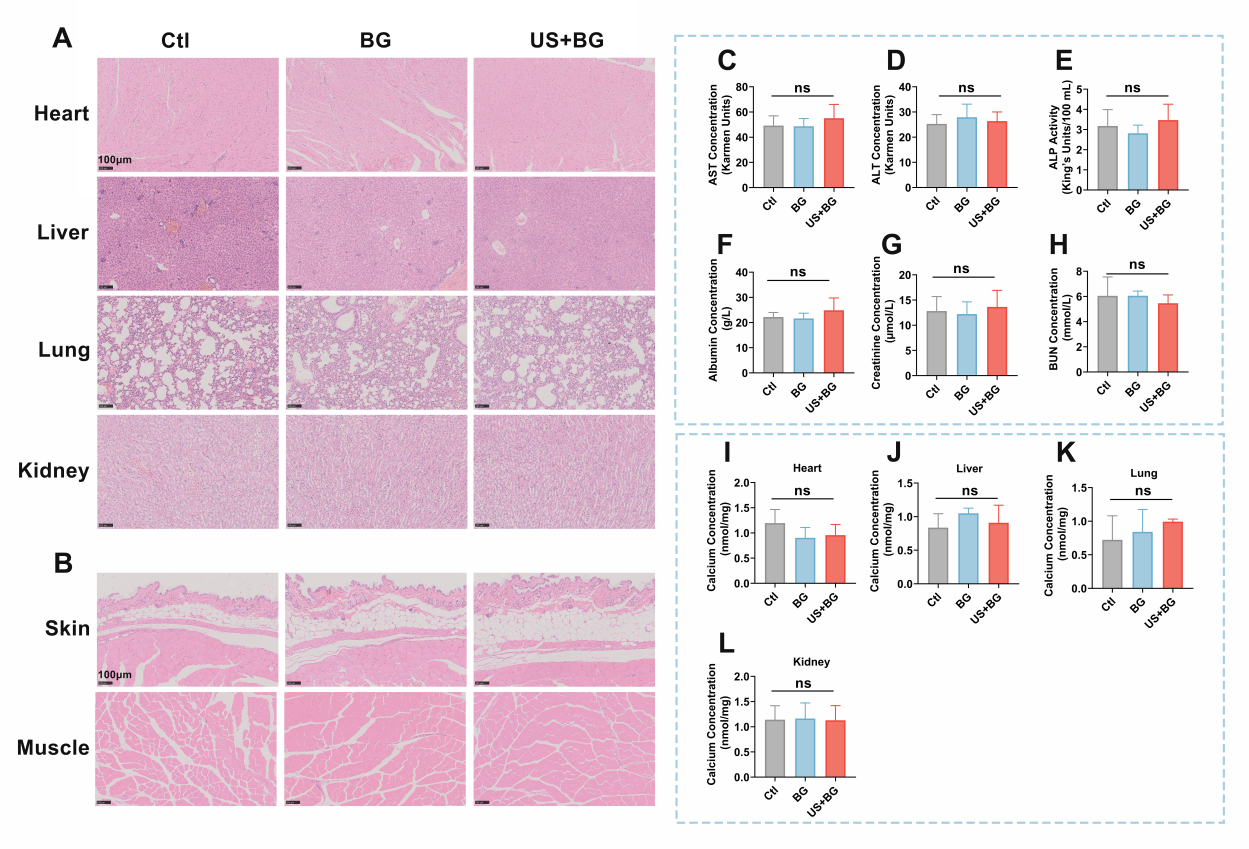


**Figure S7.** The evaluation of biosafety and calcium distribution in orthotopic breast cancer mice. (A) Representative HE staining images of the main organs (Heart, Liver, Lung, and Kidney). (B) Representative HE staining images of the skin and muscle tissues surrounding injection site. The results of liver function test by serum analysis: (C) aspartate aminotransferase (AST), (D) alanine aminotransferase (ALT), (E) alkaline phosphatase (ALP), and (F) albumin (ALB). The results show that treatment with BG and the combination of US and BG did not significantly affect liver function in mice. The results of kidney function test by serum analysis: (G) creatinine (CR) and (H) blood urea nitrogen (BUN) indicates that the treatment had no significant impact on kidney function in mice. Calcium content in (I) heart, (J) liver, (K) lung, (L) and kidney of orthotopic breast cancer mice. Data were presented as mean ± SD (n = 3). Statistical significance was assessed by one-way ANOVA followed by Tukey’s multiple comparison test. ns means no statistical difference.


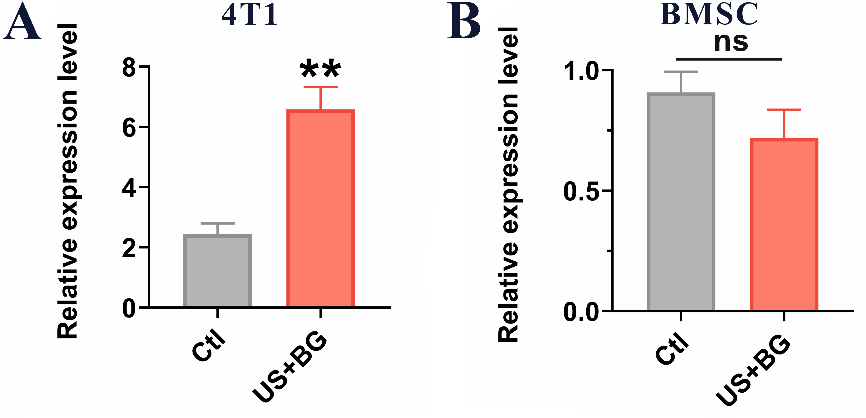


**Figure S8.** (A) The relative expression of TRPV2 in 4T1 cells. (B) The relative expression of TRPV2 in BMSC cells. Data are presented as mean ± SD (n = 4). Statistical significance was determined using a t-test. *p < 0.05, **p < 0.01.


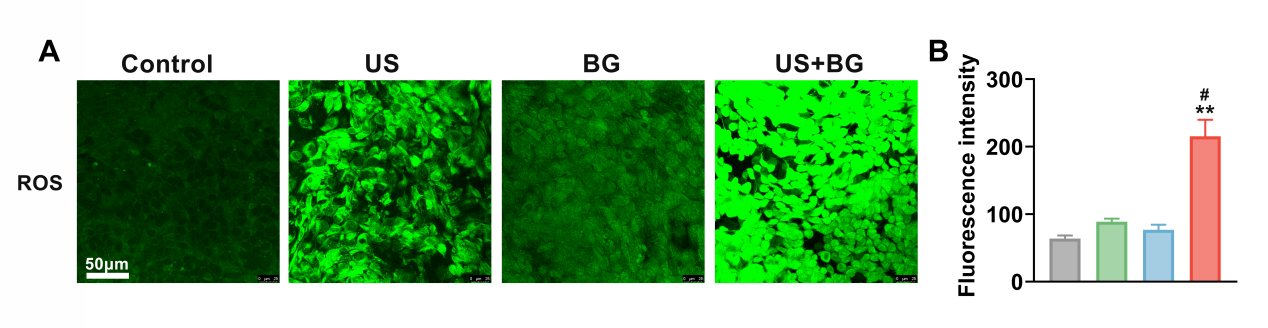


**Figure S9.** (A) Representative images of ROS in breast cancer cells after different interventions; (B) Quantification of ROS levels. Data were presented as mean ± SD (n = 3). One-way ANOVA with Tukey’s post-hoc test was used to compare multiple groups. *: Compared with the control group, *p < 0.05, **p < 0.01;#: Compared with the BG group, #p < 0.05.


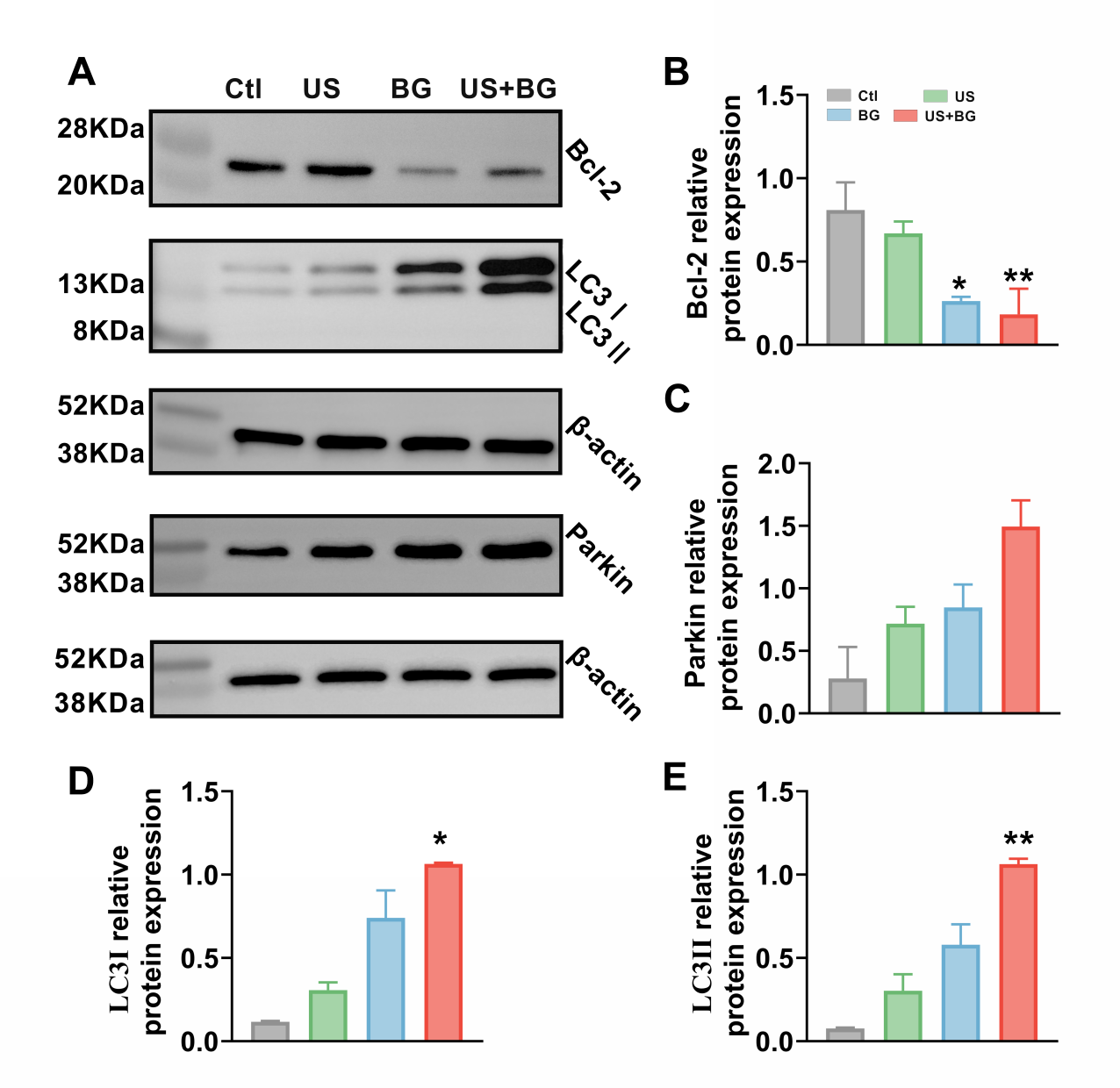


**Figure S10.** (A) Changes in the expression levels of Bcl-2, LC3Ⅰ, LC3Ⅱ, and Parkin proteins in breast cancer cells following different interventions; (B-E) Statistical charts of protein expression levels. Data were presented as mean ± SD (n = 3). One-way ANOVA with Tukey’s post-hoc test was used to compare multiple groups. *: Compared with the control group, *p < 0.05, **p < 0.01.


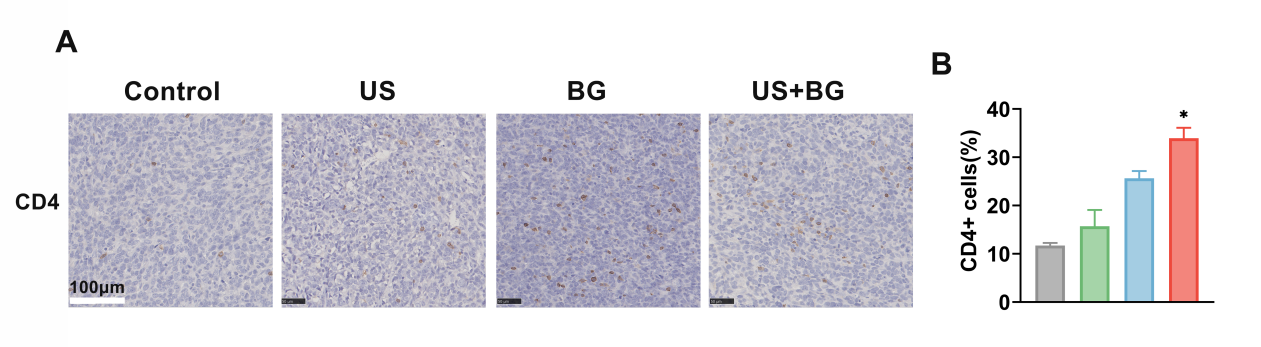


**Figure S11.** (A) Representative images of CD4 expression in tumors from an orthotopic breast cancer animal model. (B) Quantification of CD4 expression. Data were presented as mean ± SD (n = 3). One-way ANOVA with Tukey’s post-hoc test was used to compare multiple groups. *: Compared with the control group, *p < 0.05.


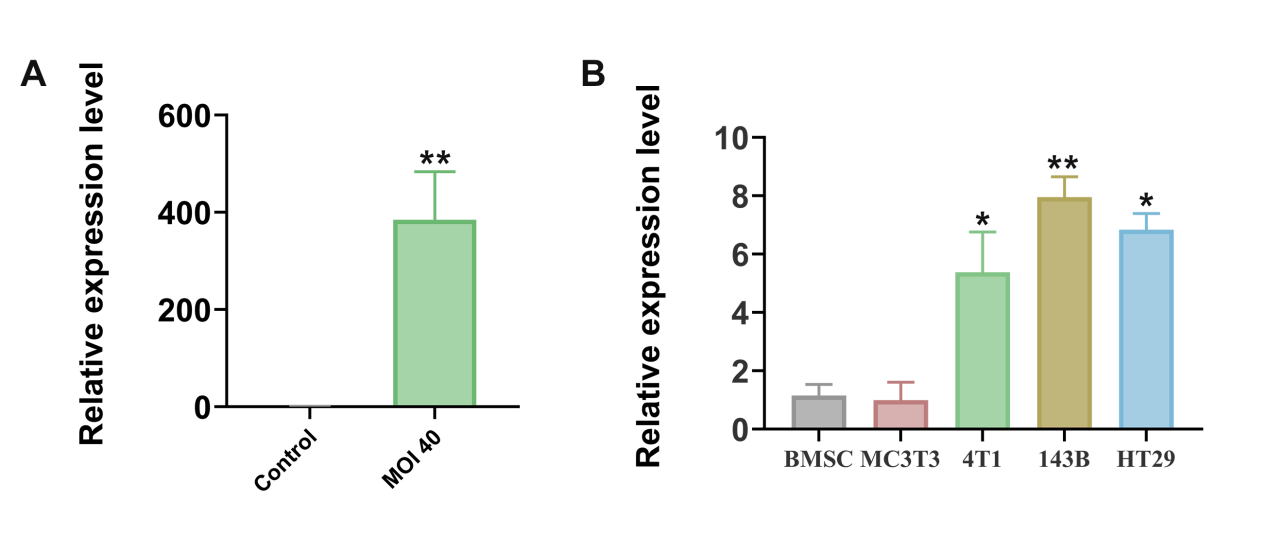


**Figure S12.** (A) Differences in TRPV2 expression between lentivirus-transduced 4T1 cells and non-transduced 4T1 cells at a multiplicity of infection of 40; (B) Differences in TRPV2 expression among different cells. Data were presented as mean ± SD (n = 3). One-way ANOVA with Tukey’s post-hoc test was used to compare multiple groups. *: Compared with the control group, *p < 0.05, **p < 0.01.


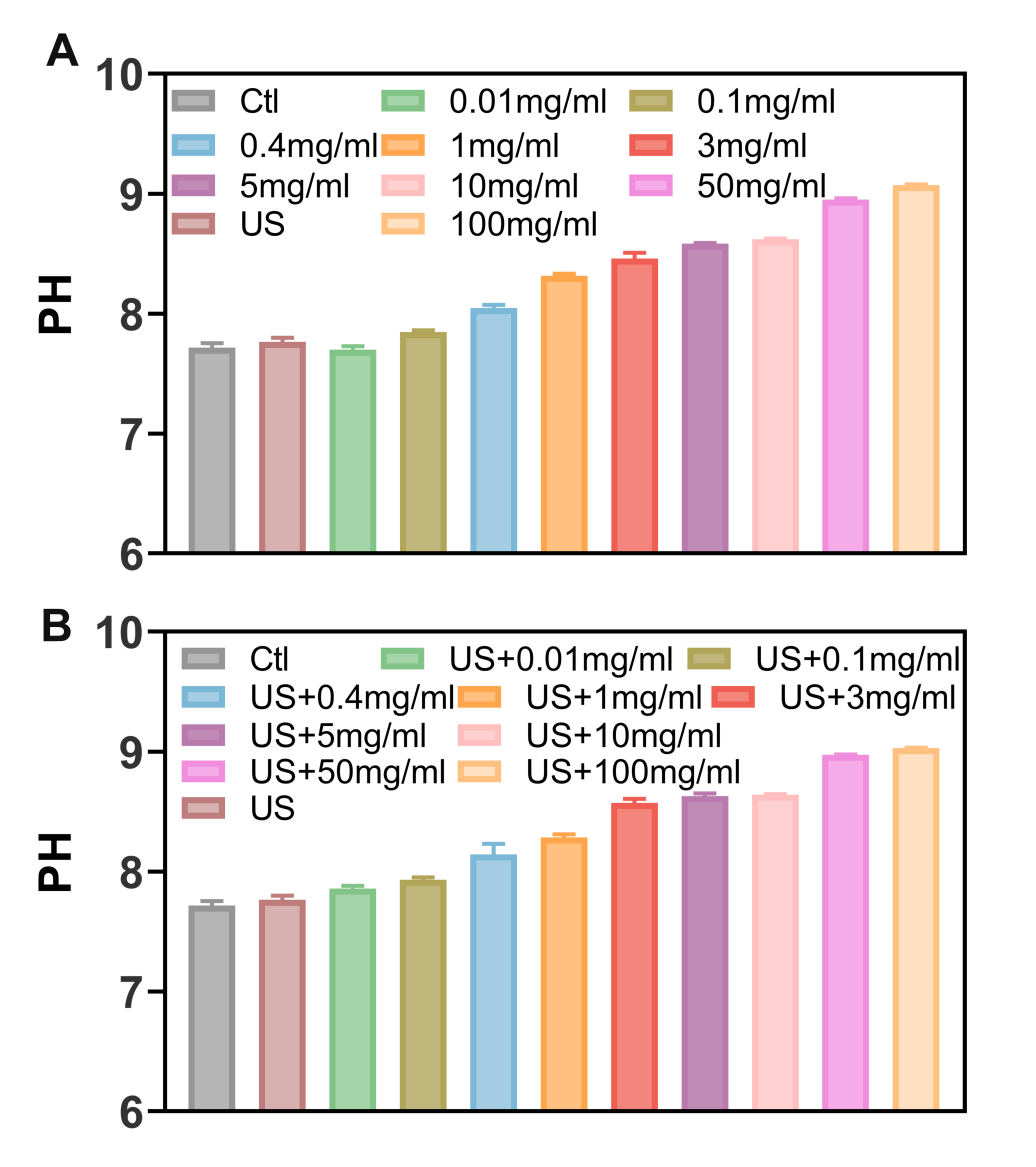


**Figure S13.** (A) pH of the culture medium after 24 hours of intervention with different concentrations of BG and cells; (B) pH of the culture medium after 24 hours of intervention with combined BG and US.


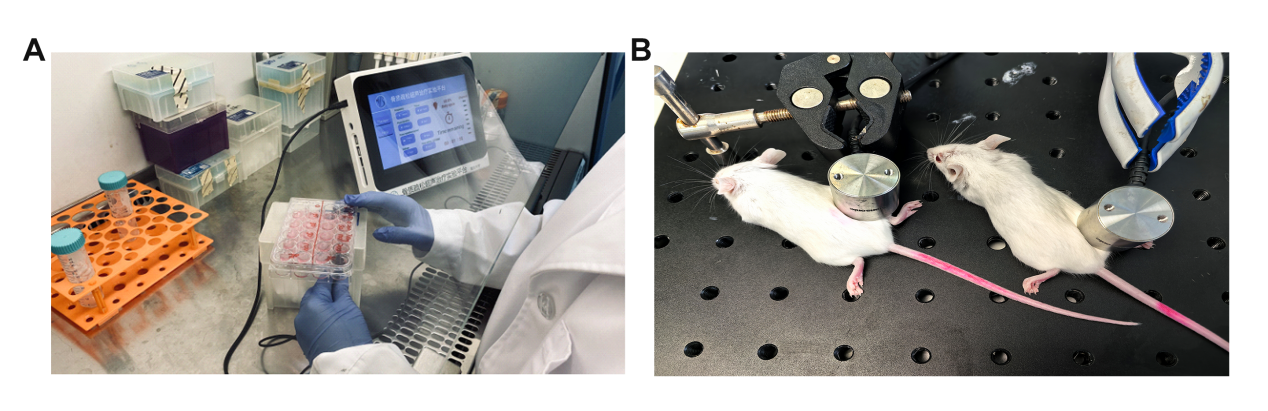


**Figure S14.** Diagrams of the ultrasound intervention procedures for cells (A) and animals (B).

Supplementary Methods

Measurement Method for Ultrasound Sound Field Distribution

The measurement of the ultrasound sound field is conducted using an ultrasound sound field distribution detection system (Acoustic Intensity Measurement System-AIMS III, ONDA Technologies). During measurement, the ultrasound probe is first fixed at a specific position in a water tank, and a cell culture plate is placed above the probe to simulate its clinical application state. A hydrophone is then used to scan the sound field point by point in the water to obtain three-dimensional sound field distribution data. The hydrophone signals are recorded by connected analytical equipment to obtain parameters such as sound pressure level, intensity, and spectrum. The measurement results of the ultrasound sound field at three different intensities are shown in Figure S2. The procedural diagrams for ultrasound intervention in cell and animal experiments can be found in Figure S14.

Table 1. Primer Sequence Information (BioTNT)

|  | Forward Primer Sequence | Reverse Primer Sequence |
| --- | --- | --- |
| TRPV2 | GCTTCGTGGAGACTGAATGGTA | TGTAGATGCCTGTGTGCTGAAA |
| GAPDH | GACACTGAGCAAGAGAGGCCCTA | TGGGATGGAAATTGTGAGGGA |
